# Epigenetic silencing of CDKN1A and CDKN2B by SNHG1 promotes the cell cycle, migration and epithelial-mesenchymal transition progression of hepatocellular carcinoma

**DOI:** 10.1038/s41419-020-03031-6

**Published:** 2020-10-02

**Authors:** Bei Li, Ang Li, Zhen You, Jingchang Xu, Sha Zhu

**Affiliations:** 1 Department of Biliary Surgery, West China Hospital of Sichuan University, Chengdu, 610041 Sichuan China; 2 Department of Pancreatic Surgery, West China Hospital of Sichuan University, Chengdu, 610041 Sichuan China; 3 Department of Urology, West China Hospital of Sichuan University, Chengdu, 610041 Sichuan China

**Keywords:** Liver cancer, Oncogenes, Cell signalling

## Abstract

Enhanced SNHG1 (small nucleolar RNA host gene 1) expression has been found to play a critical role in the initiation and progression of hepatocellular carcinoma (HCC) with its detailed mechanism largely unknown. In this study, we show that SNHG1 promotes the HCC progression through epigenetically silencing CDKN1A and CDKN2B in the nucleus, and competing with CDK4 mRNA for binding miR-140-5p in the cytoplasm. Using bioinformatics analyses, we found hepatocarcinogenesis is particularly associated with dysregulated expression of SNHG1 and activation of the cell cycle pathway. SNHG1 was upregulated in HCC tissues and cells, and its knockdown significantly inhibited HCC cell cycle, growth, metastasis, and epithelial–mesenchymal transition (EMT) both in vitro and in vivo. Chromatin immunoprecipitation and RNA immunoprecipitation assays demonstrate that SNHG1 inhibit the transcription of CDKN1A and CDKN2B through enhancing EZH2 mediated-H3K27me3 in the promoter of CDKN1A and CDKN2B, thus resulting in the de-repression of the cell cycle. Dual-luciferase assay and RNA pulldown revealed that SNHG1 promotes the expression of CDK4 by competitively binding to miR-140-5p. In conclusion, we propose that SNHG1 formed a regulatory network to confer an oncogenic function in HCC and SNHG1 may serve as a potential target for HCC diagnosis and treatment.

## Introduction

Hepatocellular carcinoma (HCC) is malignant liver cancer, which accounts for the fourth major cause of cancer-related deaths in less developed regions according to GLOBOCAN 2012^1^. It is found that about three-quarters of HCC are due to chronic hepatitis B virus (HBV) and hepatitis C virus (HCV) infection, and over 80% of HCC occur in developing countries such as sub-Saharan Africa, Southeast Asia, and East Asia^2^. Although antiviral drugs were used for HBV and HCV treatment, the treatment was far from enough. It is necessary to find more feasible methods for prevention, diagnosis, and therapy of HCC.

Long noncoding RNAs (lncRNAs) are RNA transcripts with over 200 nucleotides in length but do not code proteins^3^. Accumulating studies have revealed that the transcription and posttranscriptional controls exerted by lncRNAs play an important role in the biological development of various cancers including lung cancer and HCC^4,5^. Small nucleolar RNA host gene 1 (*SNHG1*, GenBank accession ID: 23642) has 11 exons and is found at 11q12.3. A study found that SNHG1 was found to be upregulated in non-small cell lung cancer and the overexpression promoted cell proliferation^5^. Previous clinical research defined *SNHG1* as a carcinogenic gene in HCC, which stimulates cell proliferation, promotes the course of cell cycle, and delays cell apoptosis^6^. These findings demonstrated SNHG1 was involved in certain cancer progress, but the mechanisms were left to be further studied.

MicroRNAs (miRNAs) are small, noncoding RNA transcripts with ~20 base pairs in length, which regulate miscellaneous biological activities by combining with target gene on posttranscriptional stage. Recent studies suggested that miRNAs were associated with cancer progression. For example, miR-122 was found to be downregulated in HCC tissues and enhancing miR-122 expression in HCC with or without antitumor agents could be feasibly applied to future HCC treatment strategy^7^. In addition, a study revealed that miR-140-5p targeting Nrf2 and Sirt2 accelerated doxorubicin-induced cardiotoxicity through encouraging oxidative stress in the heart^8^. Furthermore, another study reported that miR-140-5p was downregulated in breast cancer, including both primary tumor and cases with metastasis. Meanwhile, the angiogenesis and invasion of breast cancer was inhibited by exogenous expression of miR-140-5p through targeting vascular endothelial growth factor A^9^.

Cell cycle is a particularly necessary progress for the growth and development of organisms. The cell cycle of eukaryote comprises M phase and interphase, and the interphase consist of G1, S, and G2 phases^10^. Before initiating DNA replicating process, cells in G1 can enter a resting state called G0 and then stop dividing. In addition, the cell cycle control system composed of a complex network of regulatory proteins, e.g., the cyclin-dependent kinases (CDKs) participates in regulating the cell cycle progression. CDK2/4/6 controls the checkpoint progression from G1 to S phase in cell cycle. Binding of their activated subunits, the E- or A-type cyclins for CDK2 and the D-type cyclins for CDK4/6, leads to retinoblastoma protein (Rb) phosphorylation and promotes the progression of cell cycle into S phase^10^. Another research suggested that inhibiting CDK4/6 expression arrested epithelial cell cycle and alleviated acute kidney injury^11^, which indicated CDK4/6 played a crucial role in cell proliferation. A clinical research found 73% HCC samples had upregulated the expression of CDK4 protein, whereas 66% had downregulated the expression of cyclin D1 protein^12^. Therefore, CDK4 could be regarded as a clinical prognostic marker for HCC progression and it could be inferred that expressions of CDK4 were essential for certain cancer progress.

In current study, lncRNAs and mRNAs with different expression levels were screened out in HCC based on microarray analysis. Differentially expressed SNHG1, *CDK4*, and their targets were identified. SNHG1 could promote malignancy of HCC through regulating the transcription of CDKN1A and CDKN2B epigenetically in the nucleus and act as a sponge for miR-140-5p to enhance CDK4 expression in the cytoplasm. Hopefully, this study could unveil a new potential and relevant mechanism involved in HCC malignant progression, and may eventually open new promising therapeutic windows for HCC treatment.

## Results

### SNHG1 and CDK4 were highly expressed and cell cycle pathway was activated in HCC

**Fig. 1:**
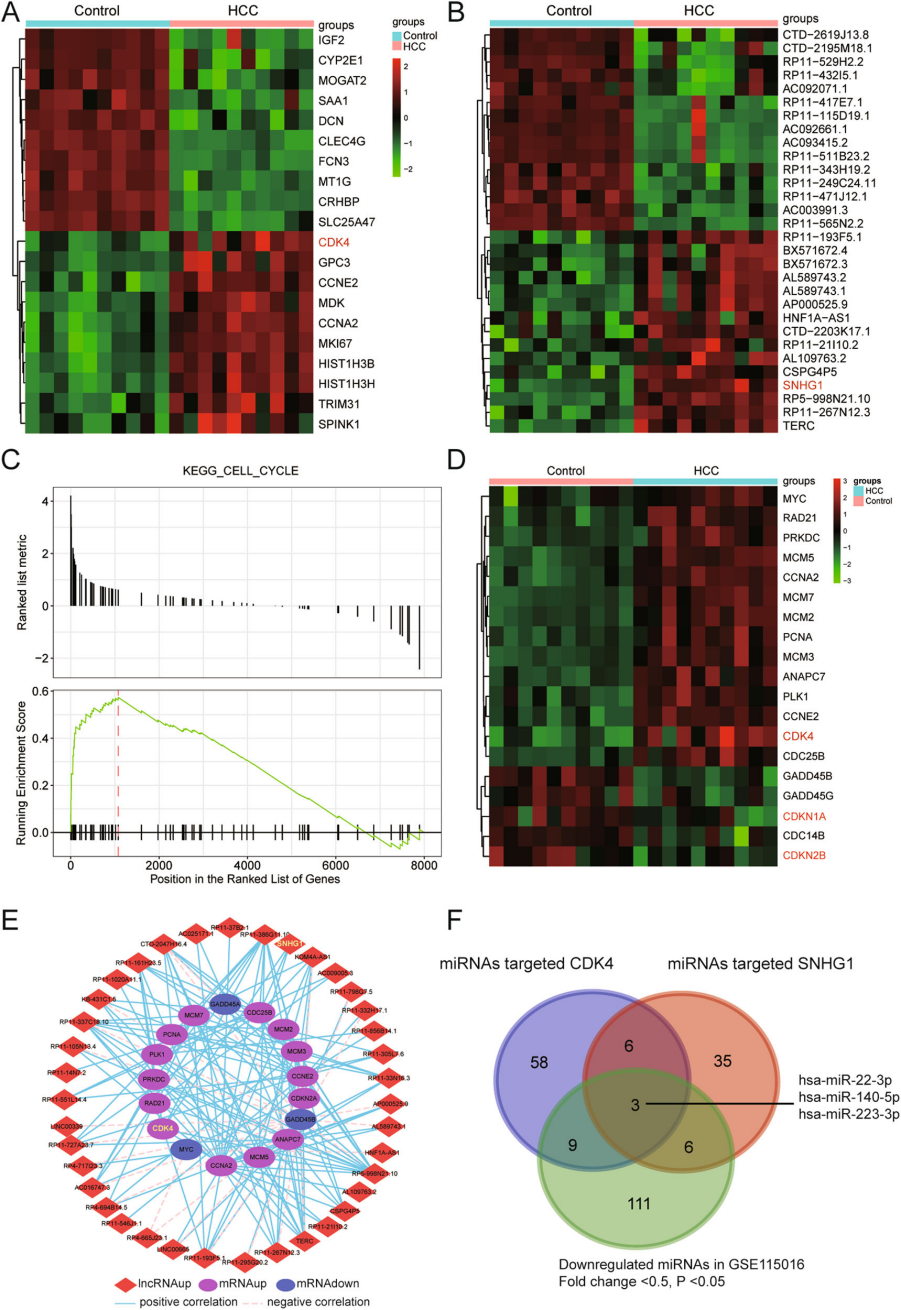
Dysregulated mRNAs, lncRNAs, and signaling pathways in HCC were identified. **a** Heatmap of top 20 differentially expressed mRNAs in HCC based on GSE115018. *CDK4* was upregulated in HCC. **b** Heatmap of top 30 differentially expressed lncRNAs in HCC based on GSE115018. SNHG1 was upregulated in HCC. Differential expression analysis was performed with *P* < 0.05 and |log2FC| > 1. **c** Gseaplot confirmed that the cell cycle pathway was activated as the running enrichment score was positive. **d** The differentially expressed genes in cell cycle pathway were revealed in heatmap and *CDK4* as one of them was upregulated in HCC. **e** Co-expression network between differentially expressed lncRNAs and mRNAs was visualized by Cytoscape (V3.6.0). Differentially expressed lncRNAs were indicated by rhombus and differentially expressed mRNAs were indicated by ellipse. Red suggested upregulation and blue meant downregulation in HCC compared with adjacent control. Blue full line and pink dotted line meant positive and negative correlation between lncRNAs and mRNAs with differential expression levels, respectively. **f** Venn gram of the intersection of target miRNAs of *CDK4* and SNHG1 predicted by TargetScan and miRanda, as well as downregulated miRNAs through bioinformatic analysis.

The expression profiling of lncRNA and mRNA on GSE115018 were downloaded from the Gene Expression Omnibus, in which a significant difference between the expression of lncRNAs and mRNAs in HCC and that of the adjacent control was discovered by microarray analysis (*P* < 0.05 and |log_2_FC| > 1). The top 20 differentially expressed mRNAs (Fig. 1a) and the top 30 differentially expressed lncRNAs (Fig. 1b) were demonstrated using heatmap, in which both *CDK4* and SNHG1 were upregulated in HCC group in comparison with the control group.

To recognize the signaling pathway involved in HCC development, KEGG (Kyoto Encyclopedia of Genes and Genomes) enrichment analysis was performed based on the microarray analysis results. Total 14 enriched KEGG pathways with significant dysregulation were ranked according to the normalized enrichment score value (Supplementary Fig. S1a). Similarly, the cell cycle pathway was displayed as an activated pathway in HCC by dotplot according to the results of gene set enrichment analysis (Supplementary Fig. S1b). Ridgeplot also demonstrated the activated cell cycle pathway (Supplementary Fig. S1c). In gseaplot analysis, the activation of cell cycle pathway was confirmed as the running enrichment score was positive (Fig. 1c). Different expression of genes involved in the cell cycle were also revealed by heatmap. *CDK4* was upregulated while *CDKN1A* and *CDKN2B* was downregulated in HCC (Fig. 1d).

To explore the relationship between lncRNAs and mRNAs with differential expression levels, co-expression network was constructed. As shown as Fig. 1e, SNHG1 was positively correlated to *CDK4*. Furthermore, nine miRNAs targeting SNHG1 and *CDK4* were predicted using TargetScan and miRanda. Meanwhile, those miRNAs underwent further cross-check with GSE115016 where the miRNA profiles of the same patients were available. Further, three out of nine stood out for their lower expression in GSE115016 and were used for further research (Fig. 1f). Those nine miRNAs were cross-validated with quantitative reverse-transcriptase PCR in HCC tissues. All of those three filtered miRNAs showed a lower expression in HCC tissues compared with normal counterparts (Supplementary Fig. S2a). With respect to the remaining six miRNAs, four miRNAs (hsa-miR-371a-5p, hsa-miR-330-5p, hsa-miR-494-3p, and hsa-miR-766) were found to be upregulated, one miRNA (hsa-miR-483-3p) was found to be downregulated, and one miRNA (hsa-miR-205-5p) was found to be of no significance in HCC tissues (Supplementary Fig. S2c). Among these, miR-140-5p was chosen for further research, for its highest fold change.

### SNHG1 was upregulated and was expressed both in the nucleus and cytoplasm

**Fig. 2:**
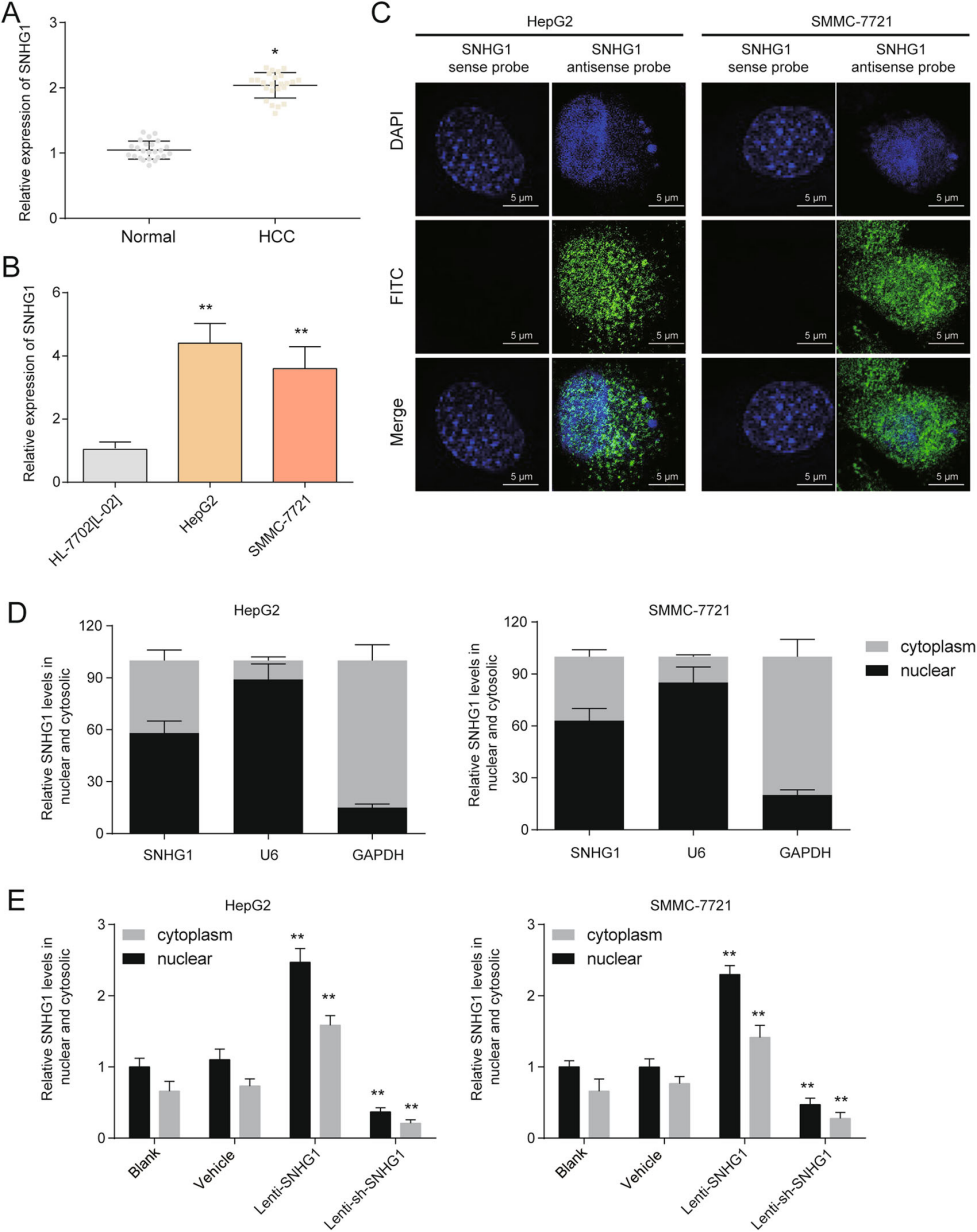
SNHG1 was upregulated in HCC and expressed both in the nucleus and cytoplasm. **a** Expressions of SNHG1 in normal tissues and HCC tissues were measured by qRT-PCR. *P < 0.05 compared with the normal tissues. **b** Expression of SNHG1 in HL-7702[L-02] (normal cells), HepG2, and SMMC-7721 (HCC cells) were measured by qRT-PCR. **P < 0.01 compared with the L-O2. **c** Representative FISH images indicated subcellular location of SNHG1 in HepG2 and SMMC-7721 (green). Nuclei were stained by DAPI (blue). SNHG1 sense probe was employed as a negative control. **d** Relative SNHG1 expression levels in nuclear and cytosolic fractions of HepG2 and SMMC-7721. Nuclear controls: U6; Cytosolic controls: GAPDH. **e** Relative SNHG1 expression levels in nuclear and cytosolic fractions of HepG2 and SMMC-7721 after SNHG1 overexpression or knockdown. **P < 0.01 compared with the vehicle group.

The expression of SNHG1 and CDK4 were further confirmed in HCC tissues and cells. Both SNHG1 and CDK4 were upregulated in HCC samples compared with normal tissues (Fig. 2a and Supplementary Fig. S2c). Correlation analysis revealed that miR-140-5p expression is negatively associated with both SNHG1 and CDK4, and SNHG1 expression is positively associated with CDK4 in 24 HCC tissues (Supplementary Fig. S2d–f). Expression level of SNHG1, miR-140-5p, and CDK4 expression was also measured in HCC cells. SNHG1 and CDK4 were found to be upregulated, whereas miR-140-5p was found to be downregulated in HepG2 and SMMC-7721 (Fig. 2b and Supplementary Fig. S2g, h). Then, we examined the subcellular localization of SNHG1 given that the function of one lncRNA depended on its subcellular distribution^13^. Through fluorescence in situ hybridization (FISH) and subcellular fractionation assays, SNHG1 was found to be expressed both in the nucleus and cytoplasm, and a larger proportion of SNHG1 was observed in the nucleus (Fig. 2c, d). Besides, both nuclear and cytosolic SNHG1 were decreased with lenti-sh-SNHG1 infection. Lenti-SNHG1 infection notably upregulated the levels of SNHG1 both in the nucleus and cytoplasm as shown in Fig. 2e.

### SNHG1 affects cell cycle, growth, migration, and epithelial–mesenchymal transition of HCC cells in vitro

**Fig. 3:**
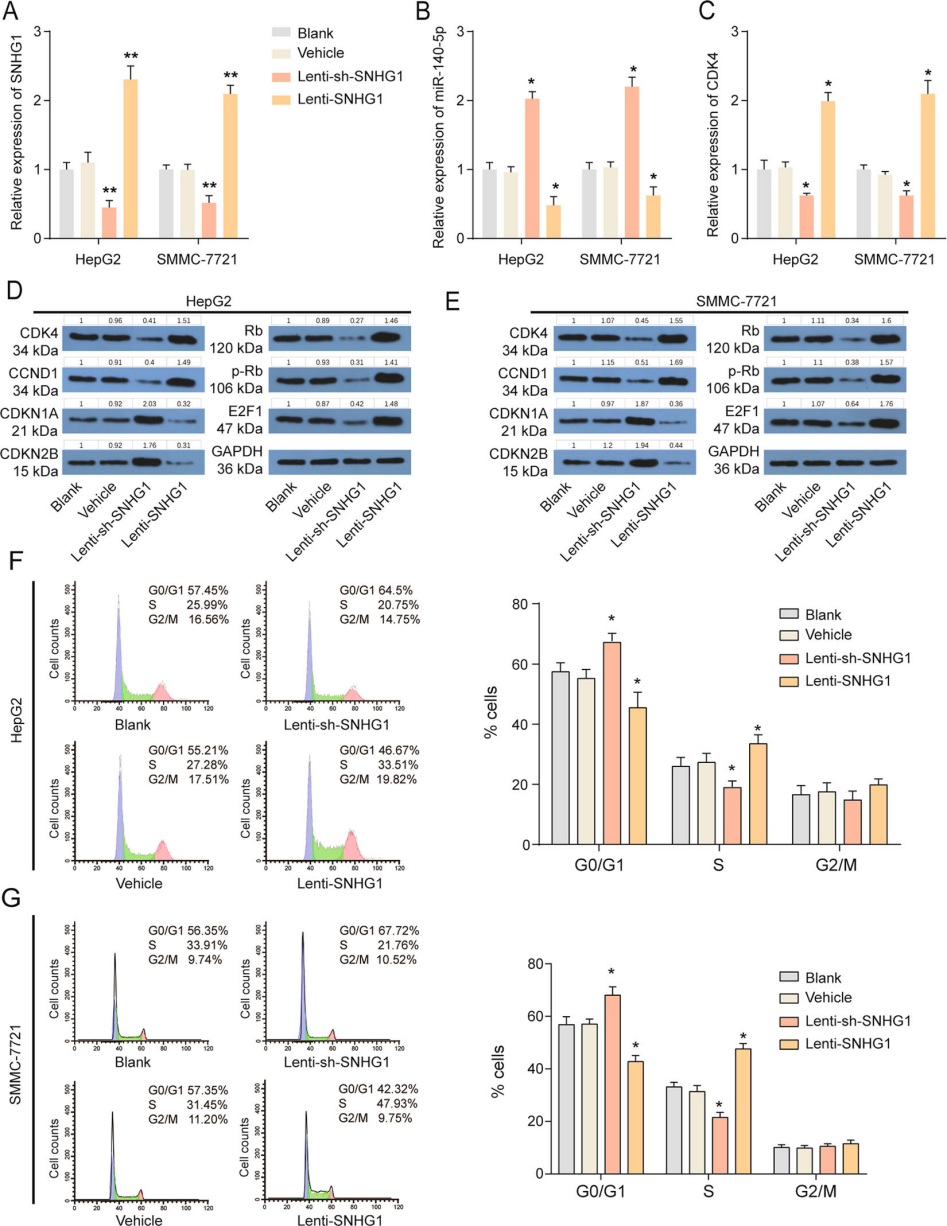
SNHG1 regulates HCC cell cycle progress. **a**–**c** Expressions of SNHG1 (**a**), miR-140-5p (**b**), and *CDK4* (**c**) in HepG2 and SMMC-7721 after indicated treatment were measured by qRT-PCR. **d**, **e** Expressions of cell cycle-related protein such as CDK4, CCND1, CDKN2A, CDKN2B, Rb, p-Rb, and E2F1 in HepG2 (**d**) and SMMC-7721 (**e**) after indicated treatment were measured by western blotting. **f**, **g** Flow cytometric cell cycle distribution assays to detect the proportion of HepG2 (**f**) and SMMC-7721 (**g**) cells in G0/G1, S, and G2/M phases after indicated treatment. *P < 0.05, **P < 0.01 compared with Vehicle.

To explore the effect of SNHG1 in HCC cells, SNHG1 overexpression and knockdown stable HCC cells were established through lentivirus-mediated stable transfection. As shown in Fig. 3a, SNHG1 expression was strongly reduced after lenti-sh-SNHG1 treatment and SNHG1 expression was strongly enhanced after lenti-SNHG1 treatment. In addition, miR-140-5p was found to be downregulated and *CDK4* mRNA was found to be upregulated after SNHG1 overexpression (Fig. 3b, c). Precise regulation of the cell cycle is necessary to ensure genomic stability and cell cycle disorders are the basic characteristics of tumor cells^14^. Then, we examined cell cycle-related proteins by western blot. The results revealed that CDK4, CCND1, Rb, p-Rb, and E2F1 were all decreased, whereas CDKN1A and CDKN2B were increased in sh-SNHG1-transfected HCC cells (Fig. 3d, e). SNHG1 overexpression had an opposite function on the expression of cell cycle-related proteins compared with SNHG1 knockdown. Furthermore, flow cytometry cell cycle analysis revealed that SNHG1 knockdown increased the proportion of cells in G0/G1 phase and decreased the proportion of cells in S phase both HepG2 and SMMC-7721 (Fig. 3f, g). Malignant tumors are always characterized by abnormal proliferation and the inhibition of cell growth is one of the approaches to tumor therapy^15^. We also detected the function of SNHG1 on HCC cell growth by MTT (3-(4,5-dimethylthiazol-2-yl)-2,5-diphenyltetrazolium bromide) assays and the result suggested that SNHG1 knockdown inhibited cell growth significantly and SNHG1 overexpression promoted cell growth (Supplementary Fig. S3a, b).

The metastasis of malignant tumors is often the main reason for the failure of tumor treatment^16^ and we further explored the role of SNHG1 in HCC metastasis. Wound-healing assay indicated that SNHG1 overexpression promoted migration and SNHG1 knockdown impaired migration of HepG2 and SMMC-7721 (Supplementary Fig. S3c, d). Transwell invasion assay showed that cell invasive ability of HepG2 and SMMC-7721 was enhanced by the overexpression of SNHG1, while impaired by downregulation of SNHG1 (Supplementary Fig. S3e, f). As epithelial–mesenchymal transition (EMT) plays an important role in the progression and metastasis of HCC^17^, we then explored the effect of SNHG1 on EMT relative protein E-cadherin, N-cadherin, and Vimentin. The results suggest that SNHG1 obviously promoted N-cadherin and Vimentin expression, and inhibited E-cadherin expression (Supplementary Fig. S3g, h). In short, SNHG1 affects cell cycle, growth, metastasis, and EMT of HepG2 and SMMC-7721.

### SNHG1 promote the growth of SMMC-7721 in vivo

**Fig. 4:**
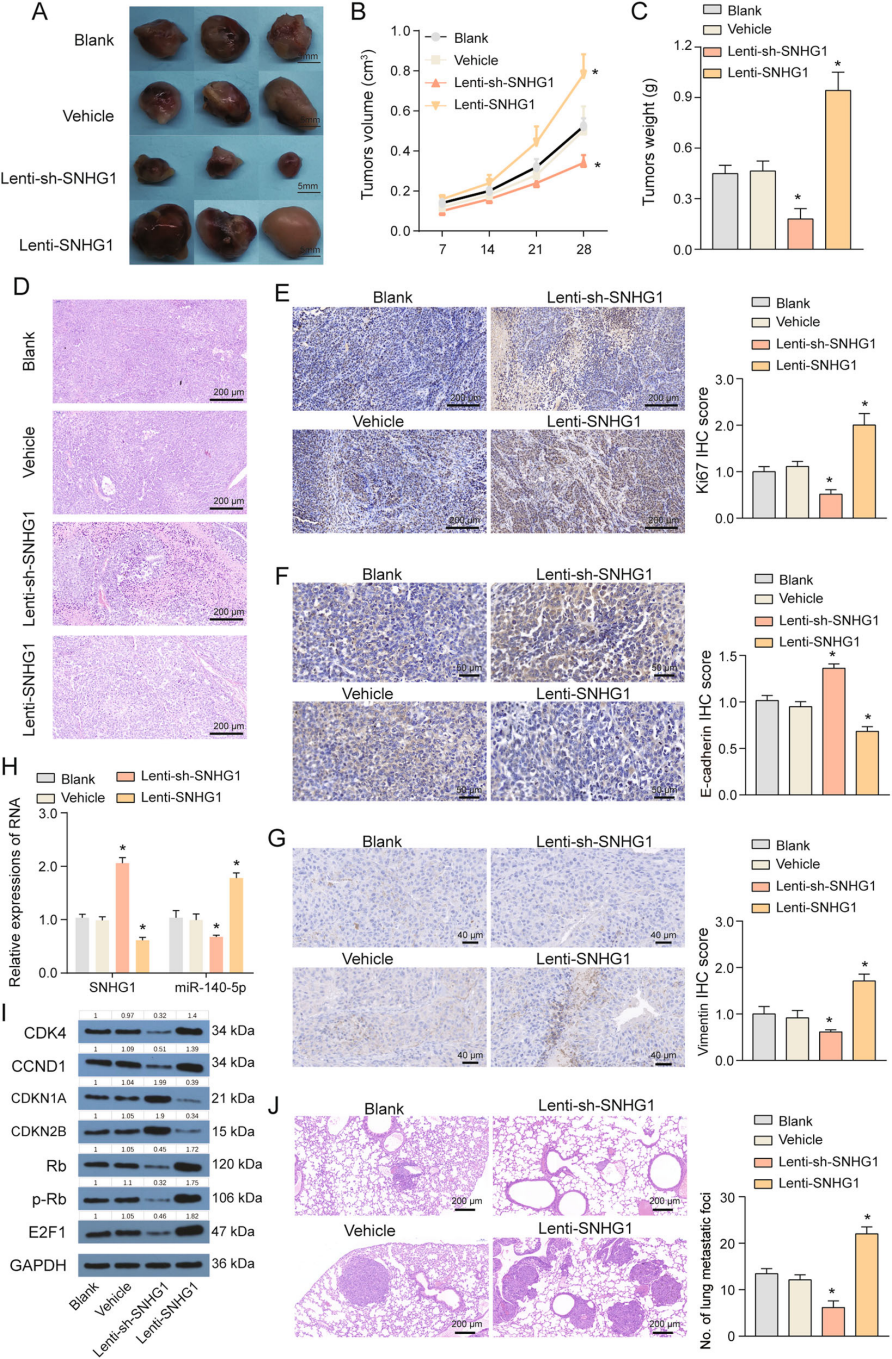
SNHG1 affects HCC growth in vivo. **a** Representative images of solid tumor after indicated treatment. **b** Tumor volume growth curves after indicated treatment. **c** Tumor weights in different groups. **d** Representative images of hematoxylin and eosin (HE) staining of tumor samples from different groups. **e**–**g** Representative images of Ki67 (**e**), E-cadherin (**f**), and Vimentin (**g**) immunostaining of tumor samples from different groups. **h** Expressions of SNHG1 and miR-140-5p in tumor samples from different groups were measured by qRT-PCR. **i** Expressions of CDK4, CCND1, CDKN2A, CDKN2B, Rb, p-Rb, and E2F1 in tumor samples from different groups were measured by western blot. **j** Representative images of HE staining of lung tissues from nude mice after tail intravenous injection with indicated SMMC-7721 cells. *P < 0.05, **P < 0.01 compared with Vehicle.

To determine whether SNHG1 could influence HCC tumorigenesis in vivo, SMMC-7721 stably transfected with sh-SNHG1, SNHG1, or empty vector were subcutaneously injected into nude mice. Twenty-eight days after the injection, tumors from the sh-SNHG1 group were significantly smaller and tumors from the SNHG1 overexpression group were significantly larger compared with the vehicle group (Fig. 4a–c). Hematoxylin and eosin (HE) staining suggested that there were large necrotic areas in sh-SNHG1 group, indicating the tumor inhibition of SNHG1 knockdown (Fig. 4d). Besides, tumor tissues collected from the sh-SNHG1 group exhibited lower Ki67-positive rates, whereas the SNHG1 group exhibited higher Ki67-positive rates compared with the control group (Fig. 4e). E-cadherin and Vimentin staining results suggested that SNHG1 promoted EMT process of SMMC-7721 in vivo (Fig. 4f, g). We also performed quantitative PCR analyses to measure SNHG1 expression in xenografted tumor tissues. As expected, solid tumor from sh-SNHG1 group exhibited reduced SNHG1 expression, whereas solid tumor from SNHG1 overexpression group exhibited increased SNHG1 expression (Fig. 4h). Meanwhile, miR-140-5p, CDKN1A, and CDKN1B were upregulated, whereas CDK4, CCND1, Rb, p-Rb, and E2F1 were downregulated in solid tumor formed form sh-SNHG1 group. However, miR-140-5p, CDKN1A, and CDKN1B were downregulated, whereas CDK4, CCND1, Rb, p-Rb, and E2F1 were upregulated in solid tumor formed from SNHG1 overexpression group (Fig. 4h, i). In addition, the result of lung metastasis assay in which SMMC-7721 cells were inoculated into the tail veins of nude mice, suggested that SNHG1 overexpression had positive effects on lung metastasis (Fig. 4j). In summary, SNHG1 promote the tumorigenesis, metastasis and EMT of SMMC-7721 in vivo.

### SNHG1 promoted HCC cells growth, migration, and invasion through miR-140-5p and CDK4

**Fig. 5:**
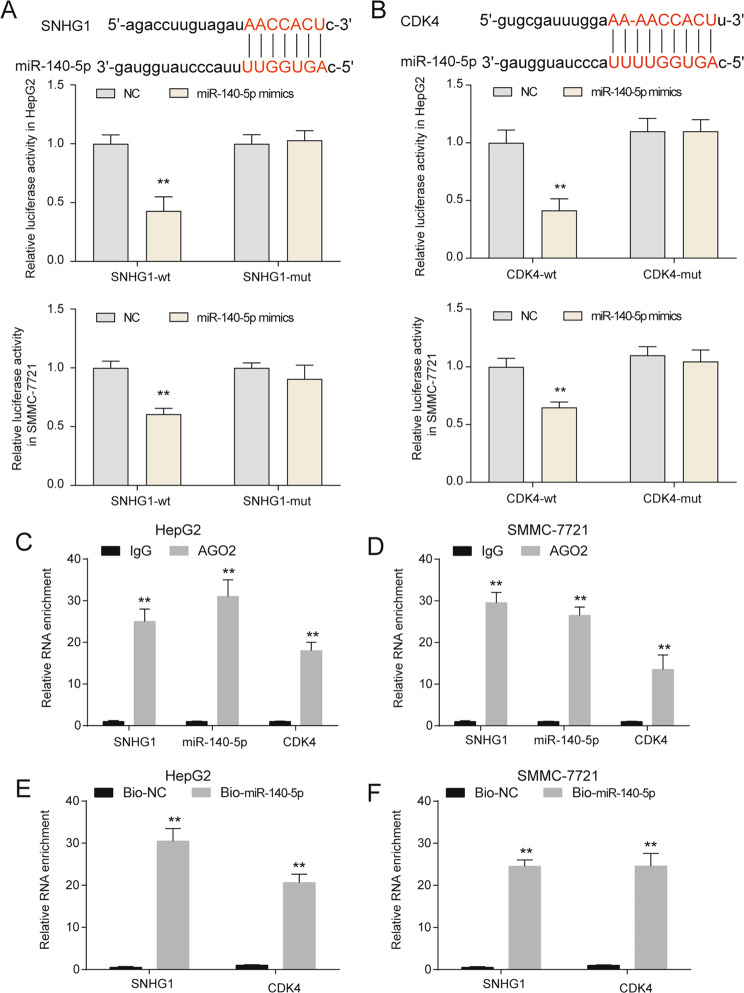
Validation of interaction between SNHG1 and miR-140-5p as well as interplay between miR-140-5p and CDK4. **a** The potential binding sequence was shown and luciferase assay was used to prove the direct combination of SNHG1 and miR-140-5p in HepG2 and SMMC-7721. **b** The potential binding sequence was shown and luciferase assay was used to prove the direct combination of miR-140-5p and *CDK4* in HepG2 and SMMC-7721. **P < 0.01 compared with NC group. **c**, **d** RNA immunoprecipitation with an anti-AGO2 antibody was used to assess endogenous Ago2 binding to RNA in HepG2 (**c**) and SMMC-7721 (**d**), IgG was used as the control. SNHG1, miR-140-5p, and CDK4 levels were determined by qRT-PCR. **P < 0.01 compared with IgG group. **e**, **f** Pulldown with a biotinylated miR-140-5p to assess miR-140-5p binding RNA in HepG2 (**e**) and SMMC-7721 (**f**), biotinylated scramble RNA was used as the control. SNHG1 and CDK4 levels were determined by qRT-PCR. **P < 0.01 compared with Bio-NC group.

Many cytoplasmic lncRNAs have been reported to act as competing endogenous RNAs by competitively binding miRNAs. miR-140-5p was predicted a target of SNHG1 using miRanda. Then dual-luciferase reporter assays were performed to determine whether miR-140-5p could directly interact with SNHG1 in HepG2 and SMMC-7721. miR-140-5p overexpression significantly reduced the luciferase activity (Fig. 5a). Similarly, we also verified the miR-140-5p also targeted to CDK4 3’UTR (Fig. 5b). In addition, RNA binding protein immunoprecipitation (RIP) assay results revealed that SNHG1, miR-140-5p and CDK4 were significantly enriched in AGO2-containing complexes, suggesting that the AGO2 protein bound to SNHG1, miR-140-5p and CDK4 directly in HepG2 and SMMC-7721 (Fig. 5c, d). Furthermore, RNA pull-down assays also confirmed that SNHG1 and CDK could be obviously enriched by Bio-miR-140-5p (Fig. 5e, f). Above all, we confirmed that miR-140-5p was a shared target of SNHG1 and CDK4, and SNHG1 promoted CDK4 expression through sponging miR-140-5p.

To investigate whether miR-140-5p and CDK4 were involved in the SNHG1 induced promotion of HCC cells growth, cell cycle, migration, and invasion, rescue assays were performed. MTT assays indicated that CDK4 overexpression or miR-140-5p inhibitor partially abolished SNHG1 knockdown induced growth inhibition of HepG2 and SMMC-7721 (Supplementary Fig. S4a, b). Flow cytometry cell cycle analysis revealed that CDK4 overexpression or miR-140-5p inhibitor partially abolished the G0/G1 arrest of HepG2 and SMMC-7721 induced by SNHG1 knockdown (Supplementary Fig. S4c, d). Similarly, CDK4 overexpression or miR-140-5p inhibitor partially rescued the inhibition of migration and invasion on HepG2 and SMMC-7721 induced by SNHG1 knockdown (Supplementary Fig. S4e, f). Above all, SNHG1 affected HCC cells growth, cell cycle, migration, and invasion through miR-140-5p and CDK4.

### SNHG1 is required for the epigenetic repression of CDKN1A and CDKN2B by interacting with EZH2

**Fig. 6:**
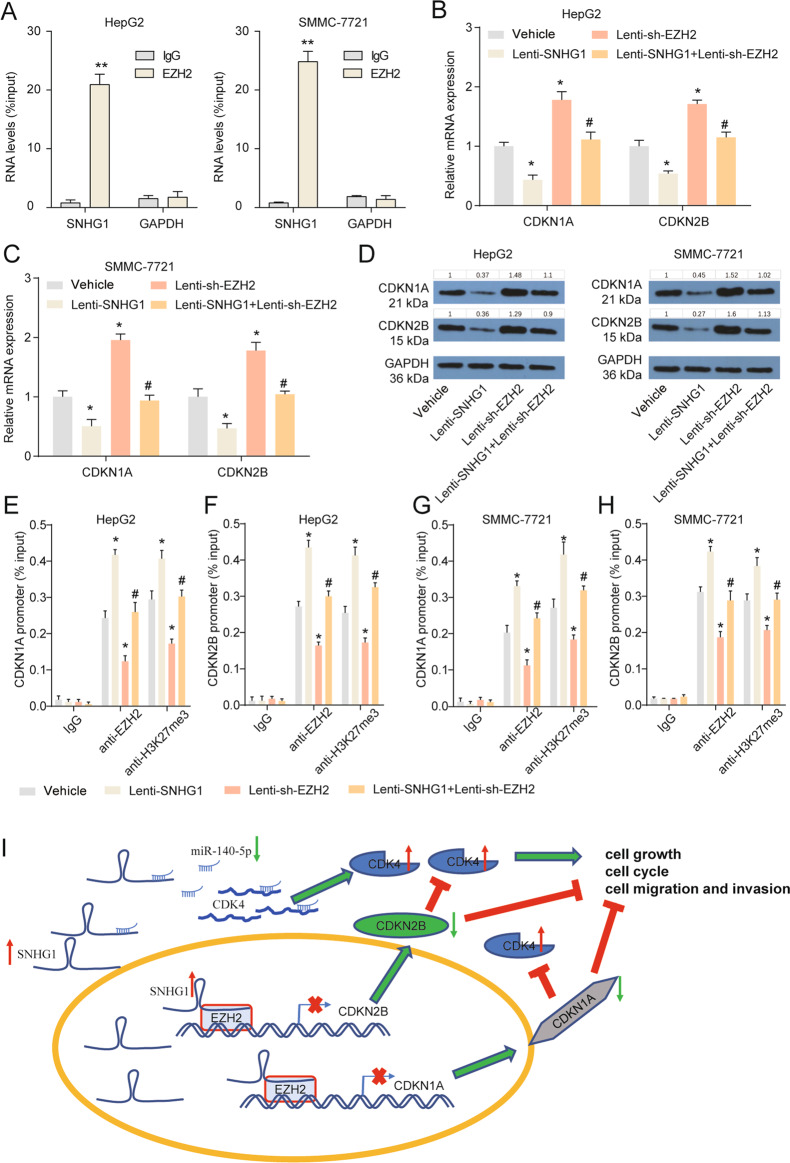
SNHG1 participates in epigenetic repression of CDKN1A and CDKN2B by interacting with EZH2. **a** RNA immunoprecipitation with an anti-EZH2 antibody was used to assess endogenous EZH2 binding to RNA in HepG2 and SMMC-7721, IgG was used as the control. SNHG1 levels were determined by qRT-PCR and GAPDH was employed as a negative control. **P < 0.01 compared with IgG group. **b**, **c** Expressions of CDKN1A and CDKN2B in HepG2 (**b**) and SMMC-7721 (**c**) after indicated treatment were measured by qRT-PCR. **d** Expressions of CDKN1A and CDKN2B in HepG2 and SMMC-7721 after indicated treatment were measured by western blotting. **e**, **f** ChIP assays were performed to detect EZH2 and H3K27me3 occupancy in the CDKN1A (**e**) and CDKN2B (**f**) promoter region in HepG2. **g**, **h** ChIP assays were performed to detect EZH2 and H3K27me3 occupancy in the CDKN1A (**g**) and CDKN2B (**h**) promoter region in SMMC-7721. *P < 0.05 compared with vehicle, #*P* < 0.05 compared with Lenti-SNHG1 group. **i** Schematic of the proposed mechanism of SNHG1 in HCC. In the cytoplasm, SNHG1 acts as a ceRNA to sponge miR-140-5p and upregulated the expression of CDK4. In the nucleus, SNHG1 is involved in EZH2 mediated epigenetic repression of CDKN1A and CDKN2B. Besides, CDKN1A and CDKN2B are well-studied inhibitor of CDK4. Downstream genes of SNHG1 formed a regulatory network to regulate cell cycle, growth, migration, and invasion of HCC.

We next explored SNHG1 functions in the nucleus. Previous studies have demonstrated that some lncRNAs in the nucleus could participate in the recruitment of EZH2 to its target genes and influence their expression^18^. EZH2 is a histone methyltransferase that can catalyze histone H3 lysine 27 trimethylation (H3K27me3) and epigenetically silence target genes^19^. We next analyzed chromatin immunoprecipitation (ChIP)-sequencing data of HepG2 downloaded from the Encyclopedia of DNA Elements (ENCODE) database^20^. H3K27me3 was highly enriched in the CDKN1A and CDKN2B promoter region (Supplementary Fig. S5a). Thus, we then explored whether there was interaction between SNHG1 and proteins of EZH2 by RIP assays and found that SNHG1 bound with EZH2 in naïve HCC cells (HepG2 and SMMC-7721) (Fig. 6a). We further found that there was a strong binding between SNHG1 and EZH2 in naive HCC cells and HCC primary cells in the nucleus, and the interaction between SNHG1 and EZH2 almost undetectable in the cytoplasm (Supplementary Fig. S5b, c). CDKN1A and CDKN2B were significantly downregulated in HCC cells after SNHG1 overexpression while CDKN1A and CDKN2B were obviously upregulated in HCC cells after EZH2 knockdown (Fig. 6b–d). EZH2 knockdown partially abolished SNHG1 overexpression induced CDKN1A and CDKN2B downregulation in HCC cells (Fig. 6b–d). To further address whether SNHG1 was involved in transcriptional repression through enrichment of H3K27me3 to the promoter regions of CDKN1A and CDKN2B, ChIP assays were performed. SNHG1 overexpression increased EZH2-binding ability to CDKN1A and CDKN2B promoters. EZH2 enrichment-induced H3K27me3 modifications also were increased in CDKN1A and CDKN2B promoter regions after SNHG1 overexpression (Fig. 6e–h). EZH2 knockdown partially abolished SNHG1 overexpression induced the enrichment of H3K27me3 in CDKN1A and CDKN2B promoter regions in HepG2 and SMMC-7721 (Fig. 6e–h).

To further uncover the role of EZH2 in HCC, a Y731D (tyrosine-to-aspartic acid substitution at amino acid 731) mutant of EZH2 was employed which completely impaired EZH2 catalytic activity and was unable to regulate H3K27 methylation^21^. In our study, we transiently reintroduced the expression of a wild-type and Y731D mutant of human EZH2 in HepG2 and SMMC-7721 for 48 h. The wild-type EZH2 notably increased H3K27me3 levels and suppressed CDKN1A and CDKN2B levels. However, EZH2 Y731D mutation had no effect on the expression of H3K27me3, CDKN1A and CDKN2B. We also verified that wild-type EZH2 promoted EMT progression as the upregulated N-cadherin and Vimentin expression and downregulated E-cadherin expression, whereas EZH2 Y731D mutation uncorrelated with EMT progression (Supplementary Fig. S6a). Furthermore, EZH2 overexpression promoted cell proliferation whereas Y731D mutant had no effect on HepG2 and SMMC-7721 cell proliferation (Supplementary Fig. S6b). Moreover, EZH2 knockdown partially rescued SNHG1 overexpression induced the acceleration of growth, cell cycle migration and invasion of HepG2 and SMMC-7721 (Supplementary Fig. S6c–h). Next, CDKN1A or CDKN2B overexpression partially rescued SNHG1 overexpression induced the acceleration of growth, cell cycle migration and invasion of HepG2 and SMMC-7721 (Supplementary Fig. S7). All in all, SNHG1 also could perform its functions partly depends on regulation of CDKN1A and CDKN2B expression.

### SP1 promotes SNHG1 expression in HCC

To investigate potential regulators of SNHG1 overexpression in HCC, Starbase (http://starbase.sysu.edu.cn/) and CORE (https://www.encodeproject.org/) database were employed. Transcription factor Sp1 was reported to be an upstream regulator in colorectal cancer^18^; here, we further investigated whether SP1 could regulate the expression of SNHG1 in liver cancer. Sp1 expression was significantly upregulated and positively correlated with SNHG1 expression in LIHC sequencing data from The Cancer Genome Atlas (TCGA) (Supplementary Fig. S8a, b). SP1 was highly enriched in the SNHG1 promoter region from CORE database (Supplementary Fig. S8c). In addition, SP1 knockdown via specific short hairpin RNA (shRNA) inhibited SNHG1 levels and SP1 overexpression significantly promoted SNHG1 expression both in HepG2 and SMMC-7721 cells (Supplementary Fig. S8d–g).

## Discussion

The key finding of this study is that SNHG1 plays a vital role in HCC progression. SNHG1 was found to be significantly upregulated in HCC tissues. Functional studies revealed tumorigenic roles of SNHG1 in promoting cell growth, cell cycle progression, migration, and invasion. Mechanistic investigations revealed that SNHG1 could exhibit different regulatory mechanisms in the nucleus and cytoplasm, and it could promote the development of HCC by regulating CDK4, CDKN1A, and CDKN2B expression.

Consistent with previous research, SNHG1 were upregulated in various kind of cancer. SNHG1 could promote HCC cells proliferation and cell cycle progression, while inhibit cell apoptosis^6^. Other genes of the SNHG family were found to have similar functions as well. For example, SNHG12 could facilitate the proliferation and migration of human osteosarcoma cells by up-regulating angiomotin gene expression^22^. Also, SNHG6 was reported to encourage proliferation of esophageal squamous cell carcinoma cells and prevent cell apoptosis^23^. In our study, SNHG1 promoted HCC progress by targeting miR-140-5p, subsequently promoting the expression of CDK4, and the overexpression of miR-140-5p could counteract the effects of SNHG1. Moreover, overexpression of SNHG1 was found in other cancers and modulate the expression of other miRNAs. For example, it was found that SNHG1 expression was increased in breast cancer. SNHG1 could block the differentiation of regulatory T cells through enhancing the expression of miR-448 expression and reducing that of indoleamine 2,3-dioxygenase. Consequently, the immune escape of breast cancer was achieved^24^. Another research observed a different miRNA, miR-195, was targeted by SNHG1. Further, high expressions of SNHG1 could exacerbate HCC by modulating miR-195^25^. Meanwhile, miR-195 might also act as an upstream modulator of CDK4 according to several researches. Exogenous expression of which might result in decreased expression of CDK4 and subsequent G1-phase arrest in HCC^26^ and in bladder cancer^27^. These studies indicated SNHG1 might act in multiple pathways and perform diverse functions in the development of cancers.

Our research found the overexpression of SNHG1 could decrease the proportion of cells in G0/G1 phase and increase the proportion of cell in S phase, which indicated cell cycle pathway was closely related with HCC progress. An increasing number of studies also provided solid evidence. It is reported that dysregulation of phosphatidylinositol 3-kinase (PI3K) signaling pathway has a potential oncogenic effect in HCC, and PI3K class IB was discovered to be involved in regulation of the cell cycle checkpoint and subsequently promote HCC cell proliferation^28^. TP53 was a particularly important tumor suppressor and transcription factor. Under stress conditions, *TP53* gene could encode P53 protein and P53 protein could induce cell cycle arrest, apoptosis, and senescence. Mutations of TP53, leading to uncontrolled cell cycle progression, occurred in ~10% to 50% of HCC cases^29^. These findings showed that cell cycle pathway was activated along with HCC progress and could be activated by multiple factors in HCC progress.

MiR-140-5p was characterized as a tumor suppressor in gastric cancer^30^, breast cancer^31^, and HCC^32^. EMT promote tumor cell migration and invasion, and accelerate tumor progression. In our study, we found that SNHG1/miR-140-5p/CDK4 axis promoted EMT progression in HCC. Excluding CDK4, miR-140-5p also reported to regulate LRP4 (low-density lipoprotein receptor-related protein 4) in gastric cancer^33^, TGFBR1^34^ and Slug^32^ in HCC, which further altering EMT progression. In addition, miR-140-5p also could target ORC1 (origin recognition complex 1) in cervical cancer^35^, PAK4 (p21-activated kinase 4) in oral squamous cell carcinoma^36^, and IGF1R (insulin-like growth factor-1 receptor) in clear cell renal cell carcinoma^37^ to affect cell cycle of tumor cells. In this study, we revealed that SNHG1 promotes cell cycle of HCC partly via targeting miR-140-5p/CDK4 pathway. In short, miR-140-5p could bind different targets to alter the progression of EMT, cell cycle and other biological process.

CDK4 could regulate cell cycle from G1 phase to S phase and the overexpression of CDK4 was essential for certain cancer progress. CDK4 expression was upregulated, which was reported to be correlated with HBV infection, tumor size, stage, and a poor survival rate^12^. Moreover, circular RNA hsa_circ_0016788 was highly expressed in HCC and it could activate CDK4 by targeting miR-486^38^. Recently, some breakthroughs of CDK4/6 inhibitor were made in treating breast cancer. Palbociclib, a selective inhibitor of CDK4 and CDK6 developed by Pfizer (USA), was demonstrated to be effective in a phase III study involving 521 patients who had hormone-receptor-positive metastatic breast cancer, and FDA approved its application for breast cancer in February 2015^39,40^. The successful example indicated that it was feasible to regard CDK4 as a drug target to restrain cancer development. However, CDK4 was also expressed in normal liver cells. Selectivity of drugs should be guaranteed in order to diminish the toxicity and side effects. Nowadays, CDK4/6 inhibitors are mainly applied on breast cancer therapy and there are few selective CDK4/6 inhibitors for HCC treatment.

In conclusion, this study illustrated that SNHG1 functions as an oncogene in HCC. SNHG1 promotes HCC cell growth, cell cycle progress, metastasis, and EMT through epigenetic silencing of CDKN1A and CDKN2B in the nucleus. In the cytoplasm, SNHG1 acts as a sponge for miR-140-5p to enhance CDK4 expression, thus facilitating cell growth, cell cycle progress, metastasis and EMT (Fig. 6i). SNHG1 participates in diverse regulatory mechanisms in different subcellular locations, and its downstream factors form a network to accelerate the progression of HCC. Our study suggested a strategy for targeting SNHG1 as a therapeutic target in HCC patients.

## Materials and methods

### Human tissue sample

Total 24 HCC tissues and para-carcinoma tissues samples were obtained from West China Hospital of Sichuan University between January 2018 and July 2018 in accordance with standard of the ethics committee of West China Hospital of Sichuan University. All participants were required to sign the consent form. All tissue samples were confirmed through pathological methods. The fresh samples were maintained at −80 °C liquid nitrogen for further use.

### Cell line and cell culture

HL-7702[L-02], the normal human liver cells, and HCC cells HepG2, as well as SMMC-7721 were acquired from BeNa Culture Collection (Beijing, China). HepG2 and HCCLM3 were placed in high-glucose Dulbecco’s modified Eagle’s medium (HyClone, USA) with 10% fetal bovine serum (FBS, Gibco, USA), while HL-7702 was placed in RPMI-1640 medium (Gibco, Invitrogen, Carlsbad, CA, USA) with 10% FBS separately. The cell culture was performed under the atmosphere of 5% CO_2_ at 37 °C.

### Cell transfection

Lentiviral constructs were used for stable or transient overexpression of SNHG1, CDK4, CDKN1A, and CDKN2B overexpression and knockdown of SNHG1 and EZH2 with shRNA (OBiO Technology). After 48 h, infected cells were cultured in media containing puromycin (2 μg/mL; Thermo Fisher Scientific) for 2 weeks, to select for stable expression. The sequences of shRNAs used are listed in Supplementary Table S1. MiR-140-5p mimics and inhibitors were obtained from Thermo Fisher (Waltham, MA, USA) and Lipofectamine 2000 (Invitrogen, USA) was utilized for miR-140-5p mimics and inhibitors transfection under the protocol of manufacturer. For rescue experiments, pCAG vectors encoding human wild-type EZH2 or EZH2 Y731D mutant were generated by GenePharma (Shanghai, China) and were transfected into HCC cells using Lipofectamine 2000 according to the manufacturer’s instructions.

### RNA fluorescence in situ hybridization

FISH assays were performed using Fluorescent In Situ Hybridization Kit (RiboBio, China) referring to the protocol. Fluorescein isothiocyanate (FITC)-labeled SNHG1 antisense probe and FITC-labeled SNHG1 sense probe were designed and synthesized by RiboBio (China). Cells were fixed in 4% formaldehyde for 15 min and then were permeabilized in phosphate-buffered saline (PBS) containing 0.5% Triton X-100 at 4 °C for 30 min, pre-hybridizated at 37 °C for 30 min in pre-hybridization solution. Next, probes were added in the hybridization solution and incubated with the cells at 37 °C overnight in the dark. Finally, the cells were counterstained with 4′,6-diamidino-2-phenylindole and imaged.

### Subcellular fractionation location

The separation of the nuclear and cytosolic fractions was using the PARIS Kit (Invitrogen, USA) according to the manufacturer’s instructions.

### Detection of cell cycle by flow cytometry

Cell cycle was determined by flow cytometry. Transfected cells were seeded in a 12-well plate and incubated in a humidified atmosphere with 5% CO_2_ at temperature of 37 °C for 1 day. Cells were then trypsinized and centrifuged at speed of 1000 r.p.m. for 5 min. After suspended by PBS, cells were re-centrifuged, precipitated and fixed by 70% ethyl alcohol overnight at 4 °C. Then, cells were stained by dye buffer with propidium iodide (Sigma-Aldrich, St. Louis, MO, USA) and RNAse A (Sigma-Aldrich, USA) in dark for 30 min at RT. Samples are quantified by a FACS Canto II flow cytometer (BD Biosciences, San Jose, CA, USA) and FlowJo analysis software (Version 7.2.2, TreeStar, Ashland, OR).

### Luciferase reporter assay

HepG2 and SMMC-7721 cells were planted in a 24-well plate at the density of 6 × 10^4^ cells per well. The fragments of SNHG1 and CDK4 containing wild-type or mutant binding sites of miR-140-5p were synthesized and sub-cloned into pmirGLO luciferase reporter vectors (Promega, Madison, WI, USA), respectively. After being incubated overnight, cells were subsequently transfected with constructed vector, miR-140-5p mimic or negative control by Lipofectamine 2000 (Invitrogen, USA) following the guidance of manufacturer. Transfected cells were collected 48 hours after culture. Luciferase activity was analyzed by a Dual Luciferase reporter assay kit (Promega, USA).

### RNA immunoprecipitation assay

The EZ Magna RNA immunoprecipitation Kit (Millipore, USA) was used according to the manual. The antibody used for RIP assays of EZH2 and EZH2 were obtained from Abcam (Cambridge, MA, USA).

### RNA pull-down assay

RNA pull-down assays were performed using a Magnetic RNA Protein Pull-Down Kit (Pierce, USA) following the manufacturer’s guidelines. Biotinylated miR-140-5p were synthesized by RiboBio (China).

### Chromatin immunoprecipitation assay

ChIP assays were performed using the EZ-CHIP Kit (Millipore, Billerica, MA, USA) according to the manual with slight modifications. EZH2 and H3K27me3 antibody were purchased from Abcam (Cambridge, MA, USA). The sequences of primers are listed in Supplementary Table S2.

### Animals and treatments

Five- to 6-week-old female BALB/c nude mice were purchased from Beijing Laboratory Animal Research Center (Beijing, China) and maintained in a specific pathogen-free circumstance. Animal experimental procedures acquired the admission of the Animal Care Committee of West China Hospital of Sichuan University. Mice were divided into four groups randomly. The groups are namely blank, vehicle, sh-SNHG1, and lenti-SNHG1 groups. Mice in blank group was injected with HepG2 cells without treatment. HepG2 cells transfected with empty vector, SNHG1 shRNA and lentiviral packaged SNHG1 overexpression plasmid were separately injected into the right flank of above-mentioned another three groups of mice subcutaneously. After injection for one month, tumor volumes were observed and measured by digital calipers, then calculated with the equation of volume = 1/2 × length × width^2^. At the same time, mice in four groups were put to killing and tumors were isolated for tumor weight examination and molecular expression detections. As for HCC metastasis mouse model, 5 × 10^5^ SMMC-7721 cells were injected into the lateral tail vein. The samples were embedded in paraffin for HE staining and immunohistochemistry staining.

### Statistical analysis

Statistical analysis was performed in GraphPad Prism (Version 7; La Jolla, CA, USA). Data were represented by the form mean ± SD. Differences between groups were assessed by the analysis of variance or Student’s *t*-test as appropriated. *P* < 0.05 was regarded to be statistically different.

## Supplementary information

Supplementary Table S1

Supplementary Table S2

Supplementary Figure Legends

Supplementary Figure S1

Supplementary Figure S2

Supplementary Figure S3

Supplementary Figure S4

Supplementary Figure S5

Supplementary Figure S6

Supplementary Figure S7

Supplementary Figure S8

Supplementary Materials and Methods
