# Supplementary material for: Epigenetic silencing of CDKN1A and CDKN2B by SNHG1 promotes the cell cycle, migration and epithelial-mesenchymal transition progression of hepatocellular carcinoma
Source: Cell Death Dis. 2020 Oct 2;11(10):823. doi: 10.1038/s41419-020-03031-6 (PMC7532449; doi:10.1038/s41419-020-03031-6)
Supplement: Supplementary file 1 — Supplementary Table S1 [file 41419_2020_3031_MOESM1_ESM.docx]

**Supplementary Table S1 The sequences of shRNAs**

| genes | Sequences (5’-3’) |
| --- | --- |
| Sh-SNHG1 | CACUUCGUGUCUGUUCCUCUGUAUA |
| Sh-EZH2 | GCTCCTCTAACCATGTTTACA |
| Sh-NC | GCAAACCGTGTATCAGATA |

**­­­­**
