# Supplementary material for: Epigenetic silencing of CDKN1A and CDKN2B by SNHG1 promotes the cell cycle, migration and epithelial-mesenchymal transition progression of hepatocellular carcinoma
Source: Cell Death Dis. 2020 Oct 2;11(10):823. doi: 10.1038/s41419-020-03031-6 (PMC7532449; doi:10.1038/s41419-020-03031-6)
Supplement: Supplementary file 2 — Supplementary Table S2 [file 41419_2020_3031_MOESM2_ESM.docx]

**Supplementary Table S2** **The list of primers.**

| **qPCR primers** | |  | |
| --- | --- | --- | --- |
| **genes** | Forward (5′-3’) | | Reverse (5′-3’) |
| SNHG1 | AGGCTGAAGTTACAGGTC | | TTGGCTCCCAGTGTCTTA |
| CDK4 | GCTGCTGGAAATGCTGAC | | CACTCCATTGCTCACTCC |
| CDKN1A | GGAAGGGACACACAAGAAGAAG | | AGCCTCTACTGCCACCATCTTA |
| CDKN2B | CTGGACCTGGTGGCTACG | | ACATTGGAGTGAACGCATCG |
| SP1 | TCCAGACCATTAACCTCAGTGC | | TGTATTCCATCACCACCAGCC |
| GAPDH | GCGACACCCACTCCTCCAC | | TCCACCACCCTGTTGCTGTAG |
| miR-140-5p | TGCGGCAGTGGTTTTACCCTATG | | CCAGTGCAGGGTCCGAGGT |
| miR-22-3p | AAGCTGCCAGTTGAAGAACTGTA | | GCTGTCAACGATACGCTACGTAAC |
| miR-223-3p | TGTCAGTTTGTCAAATACC | | GAACATGTCTGCGTATCTC |
| miR-766 | GCGGCCGCTATACACAGAGGATTGCTTAG | | ACGCGTCAGGCAACAGATTTC |
| miR-330-5p | GCGTCTCTGGGCCTGTGTC | | AGTGCAGGGTCCGAGGTATT |
| miR-371a-5p | ACTCAAACTGTGGGGGCACT | | CTCAACTGGTGTCGTGGAGTC |
| miR-205-5p | GCGGCGGTGTAGTGTTTCCTA | | GTGCAGGGTCCGAGGT |
| miR-494-3p | GAAACATACAC GGGAAACC | | GTGCAGGGTCCGAGGT |
| miR-483-3p | CGGGATCCCTCCTGTGCCCTCTCTCTTG | | CCGCTCGAGCTGTCCCTGAGCTTGGACTC |
| U6 | CTCGCTTCGGCAGCACA | | AACGCTTCACGAATTTGCGT |
| **ChIP primers** | |  | |
| **genes** | Forward (5′-3’) | Reverse (5′-3’) | |
| CDKN1A | CCTCCTTCTTCAGGCTTGGG | CAGGCAGCATAGGGATGGAG | |
| CDKN2B | TCTGGTAAGGGTGTGCTGTG | AAAACTCCTCTGTGGCATGTG | |

|  |  |  |
| --- | --- | --- |
|  |  |  |
