# Supplementary material for: Epigenetic silencing of CDKN1A and CDKN2B by SNHG1 promotes the cell cycle, migration and epithelial-mesenchymal transition progression of hepatocellular carcinoma
Source: Cell Death Dis. 2020 Oct 2;11(10):823. doi: 10.1038/s41419-020-03031-6 (PMC7532449; doi:10.1038/s41419-020-03031-6)
Supplement: Supplementary file 3 — Supplementary Figure Legends [file 41419_2020_3031_MOESM3_ESM.docx]

**Supplementary Figure Legends**

**Supplementary Figure S1: Dysregulated biological process distinguished through bioinformatic analysis.** (**A**) Total 14 enriched KEGG pathways that were significantly dysregulated were ranked according to the NES value. And cell cycle pathway was activated in HCC. The dysregulated signaling pathways in HCC were illustrated by dotplot (**B**) and ridgeplot (**C**) based on the GSEA results. Of which, cell cycle pathway was activated in HCC.

**Supplementary Figure S2: miR-140-5p was downregulated in HCC and CDK4 was upregulated in HCC.** (**A**) Expression of miR-140-5p, miR-22-3p and miR-223-3p was detected in normal tissue and HCC tissues using qRT-PCR. **(B)** 6 filtered miRNAs levels were verified. ^*^*P* < 0.05, ^**^*P* < 0.01 compared with the normal tissues. **(C)** CDK4 expression was detected in normal tissue and HCC tissues. ^**^*P* < 0.01 compared with the normal tissues. **(D)** The correlation between SNHG1 and miR-140-5p expression analyzed in HCC samples (n = 24, R^2^ = 0.3096, P = 0.005). **(E)** The correlation between CDK4 and miR-140-5p expression analyzed in HCC samples (n = 24, R^2^ = 0.4039, P < 0.001). **(F)** The correlation between SNHG1 and CDK4 expression analyzed in HCC samples (n = 24, R^2^ = 0.2279, P = 0.018). **(G)** Expression of miR-140-5p in HL-7702[L-02] (normal cells), HepG2 and SMMC-7721 (HCC cells) were measured by qRT-PCR. **(H)** Expression of CDK4 in HL-7702[L-02] (normal cells), HepG2 and SMMC-7721 (HCC cells) were measured. ^*^*P* < 0.05, ^**^*P* < 0.01 compared with the L-O2.

**Supplementary Figure S3: SNHG1 promoted HCC cell growth, migration and invasion. (A-B)** MTT assays demonstrated that SNHG1 knockdown inhibited HepG2 **(A)** and SMMC-7721 **(B)** growth. **(C-D)** Wound healing assay represented that cell migratory ability of HepG2 **(C)** and SMMC-7721 **(D)** was enhanced by the overexpression of SNHG1 while impaired by down-regulation of SNHG1. (**E-F**) Transwell invasion assay showed that cell invasive ability of HepG2 **(E)** and SMMC-7721 **(F)** was enhanced by the overexpression of SNHG1 while impaired by down-regulation of SNHG1. **(G-H)** The levels of EMT relative protein E-cadherin, N-Cadherin and Vimentin in HepG2 **(G)** and SMMC-7721 **(H)** with the indicated treatment. ^*^*P* < 0.05 compared with vehicle.

**Supplementary Figure S4: SNHG1 knockdown inhibited HCC cell growth, migration and invasion through miR-140-5p and CDK4. (A-B)** MTT assays demonstrated that CDK4 overexpression or miR-140-5p inhibitor could reverse growth inhibition caused by SNHG1 knockdown in HepG2 **(A)** and SMMC-7721 **(B)**. **(C-D)** Flow cytometric cell cycle distribution assays demonstrated that CDK4 overexpression or miR-140-5p inhibitor could reverse G0/G1 arrest caused by SNHG1 knockdown in HepG2 **(B)** and SMMC-7721 **(D). (E)** Wound healing assay demonstrated that CDK4 overexpression or miR-140-5p inhibitor could reverse migration inhibition caused by SNHG1 knockdown in HepG2 and SMMC-7721. **(F)** Transwell invasion assay demonstrated that CDK4 overexpression or miR-140-5p inhibitor could reverse invasion inhibition caused by SNHG1 knockdown in HepG2 and SMMC-7721. ^*^*P* < 0.05 compared with vehicle, ^#^*P* < 0.05 compared with Lenti-sh-SNHG1.

**Supplementary Figure S5: SNHG1 bind to EZH2 in nucleus.** **(A)** Analysis of H3K4me3 ChIP-seq of HepG2 cells in the CDKN1A and CDKN2B locus. **(B)** The binding of SNHG1 and EZH2 in HepG2 and SMMC-7721. **(C)** The binding of SNHG1 and EZH2 in HCC primary cells (Sample#1 and Sample#5). ^**^*P* < 0.01 compared with IgG.

**Supplementary Figure S6: SNHG1 overexpression promoted HCC cell growth, migration and invasion through EZH2.**

**(A)** The indicated protein expression after the overexpression of Y731D mutation or wild-type EZH2. **(B)** The effect of Y731D mutation or wild-type EZH2 on HCC cell proliferation. **(C-D).** MTT assays demonstrated that EZH2 knockdown could partly reverse growth promotion caused by SNHG1 overexpression in HepG2 **(C)** and SMMC-7721 **(D)**. **(E-F)** Flow cytometric cell cycle distribution assays demonstrated that EZH2 knockdown could partly reverse the change of cell cycle caused by SNHG1 overexpression in HepG2 **(E)** and SMMC-7721 **(F)**. **(G)** Wound healing assay demonstrated that EZH2 knockdown could partly reverse migration promotion caused by SNHG1 overexpression in HepG2 and SMMC-7721. **(H)** Transwell invasion assay demonstrated that EZH2 knockdown could partly reverse invasion promotion caused by SNHG1 overexpression in HepG2 and SMMC-7721. ^*^*P* < 0.05 compared with vehicle, ^#^*P* < 0.05 compared with Lenti-SNHG1.

**Supplementary Figure S7: SNHG 1 promotes HCC progression partly by regulating** **CDKN1A and CDKN2B expression. (A-B)** MTT assays demonstrated that CDKN1A or CDKN2B overexpression could partly reverse growth promotion caused by SNHG1 overexpression in HepG2 **(A)** and SMMC-7721 **(B)**. **(C-D)** Flow cytometric cell cycle distribution assays demonstrated that CDKN1A or CDKN2B overexpression could partly reverse the change of cell cycle caused by SNHG1 overexpression in HepG2 **(C)** and SMMC-7721 **(D)**. **(E)** Wound healing assay demonstrated that CDKN1A or CDKN2B overexpression could partly reverse migration promotion caused by SNHG1 overexpression in HepG2 and SMMC-7721. **(F)** Transwell invasion assay demonstrated that CDKN1A or CDKN2B overexpression could partly reverse invasion promotion caused by SNHG1 overexpression in HepG2 and SMMC-7721. ^*^*P* < 0.05 compared with vehicle, ^#^*P* < 0.05 compared with Lenti-SNHG1.

**Supplementary Figure S8: SP1 promoted SNHG1 expression in HCC.** (A) Expression of SP1 in the TCGA LIHC cohorts. (B) The correlation between SP1 and SNHG1 expression in the TCGA LIHC cohorts. (C) Analysis of SP1 ChIP-seq data of HepG2 cells in the SNHG1 locus from CORE database. (D-G) SNHG1 expression was detected by qRT-PCR in HepG2 and SMMC-7721 cells infected with SP1 shRNAs or the SP1 vector.
