# Supplementary figures and images for: Epigenetic silencing of CDKN1A and CDKN2B by SNHG1 promotes the cell cycle, migration and epithelial-mesenchymal transition progression of hepatocellular carcinoma
Source: Cell Death Dis. 2020 Oct 2;11(10):823. doi: 10.1038/s41419-020-03031-6 (PMC7532449; doi:10.1038/s41419-020-03031-6)

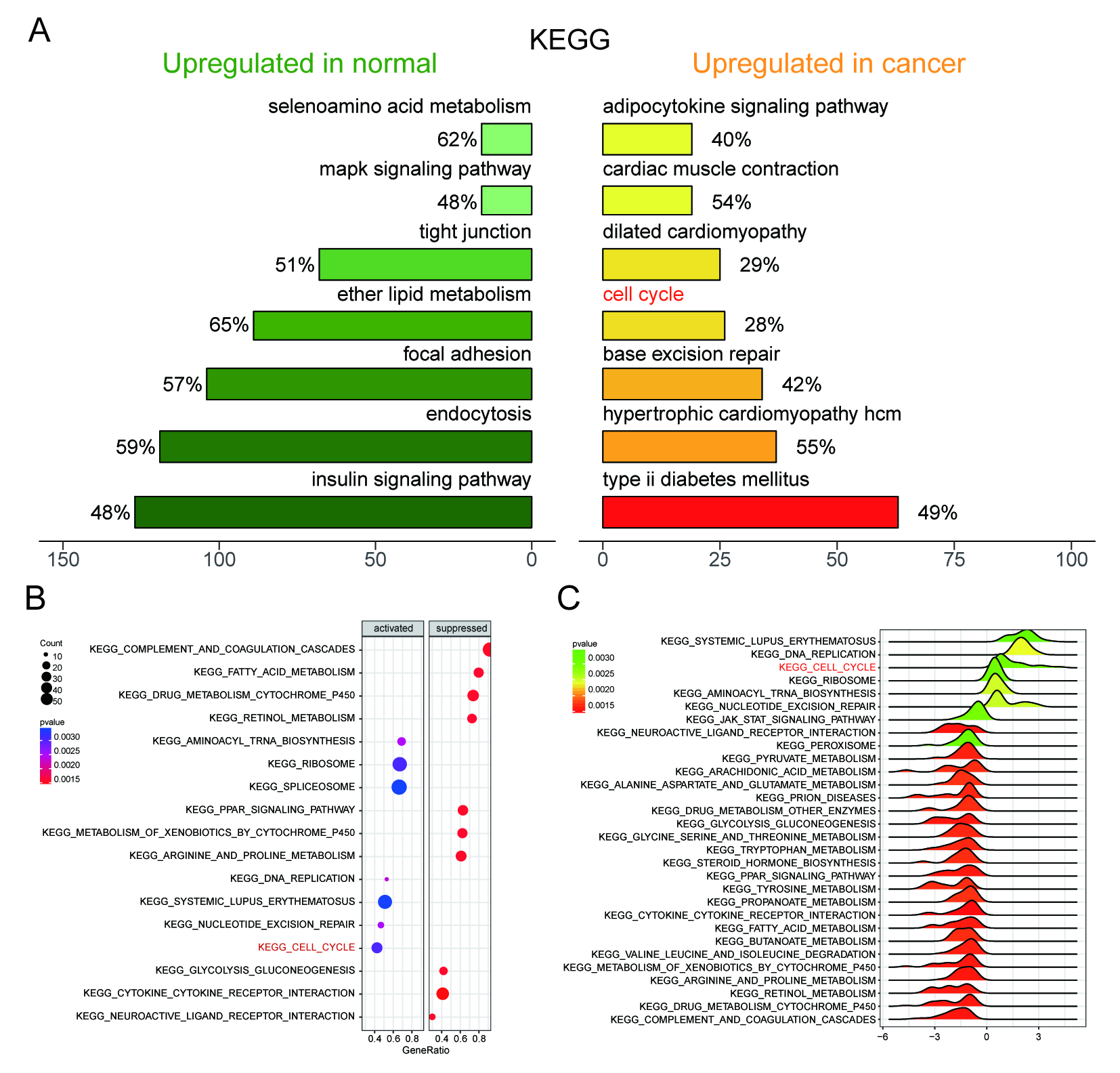

Supplement: Supplementary file 4 — Supplementary Figure S1 [file 41419_2020_3031_MOESM4_ESM.tif]

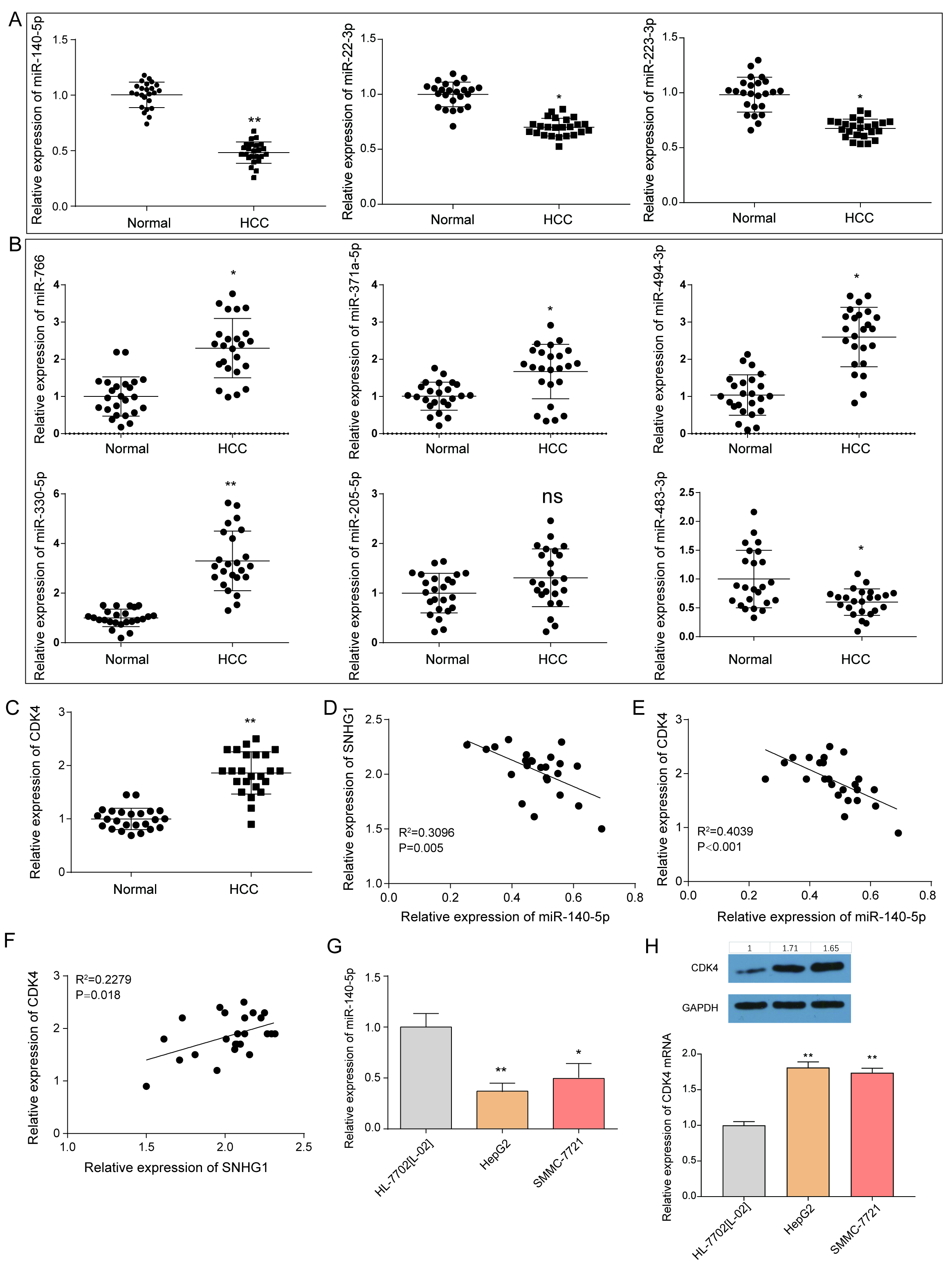

Supplement: Supplementary file 5 — Supplementary Figure S2 [file 41419_2020_3031_MOESM5_ESM.tif]

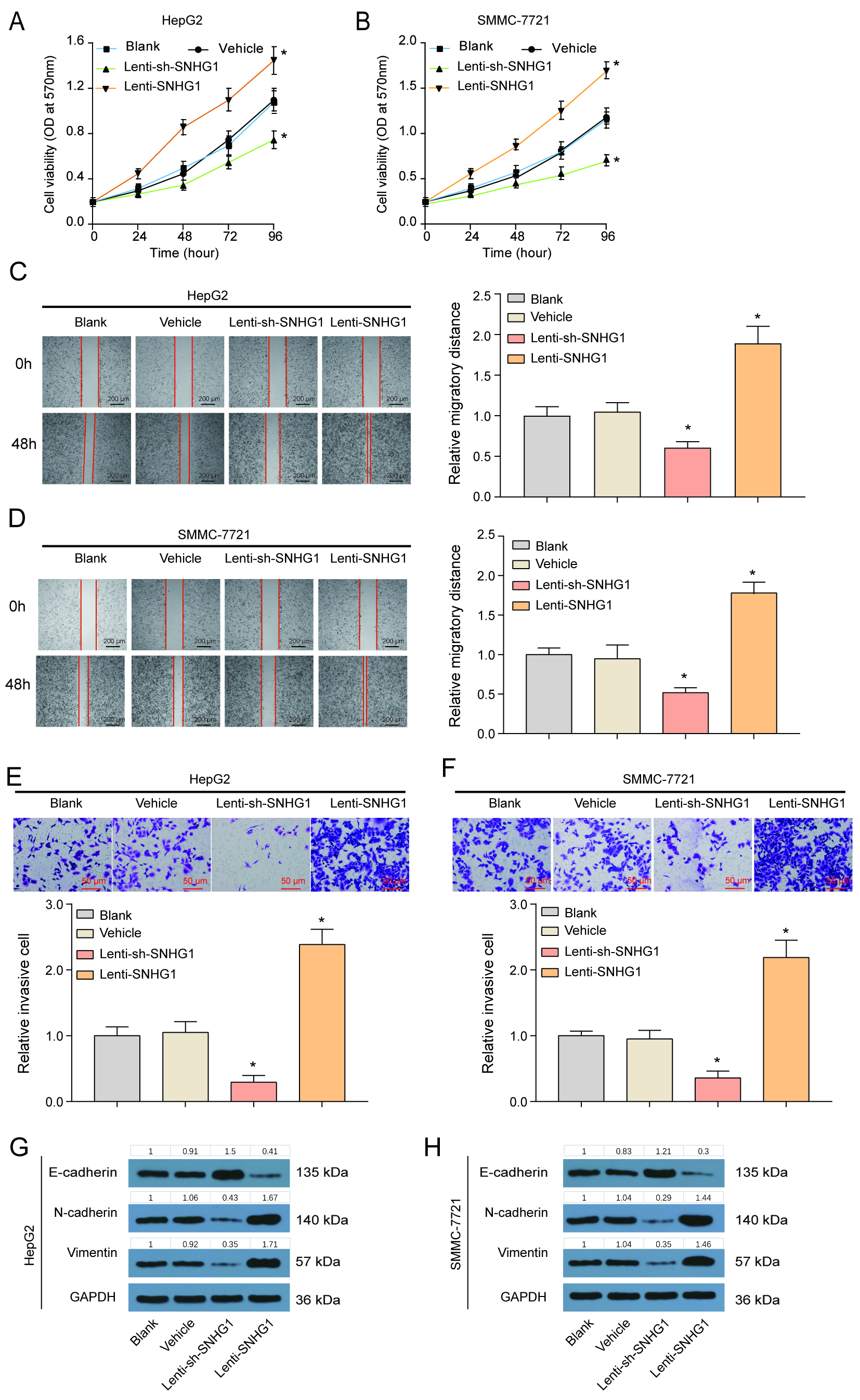

Supplement: Supplementary file 6 — Supplementary Figure S3 [file 41419_2020_3031_MOESM6_ESM.tif]

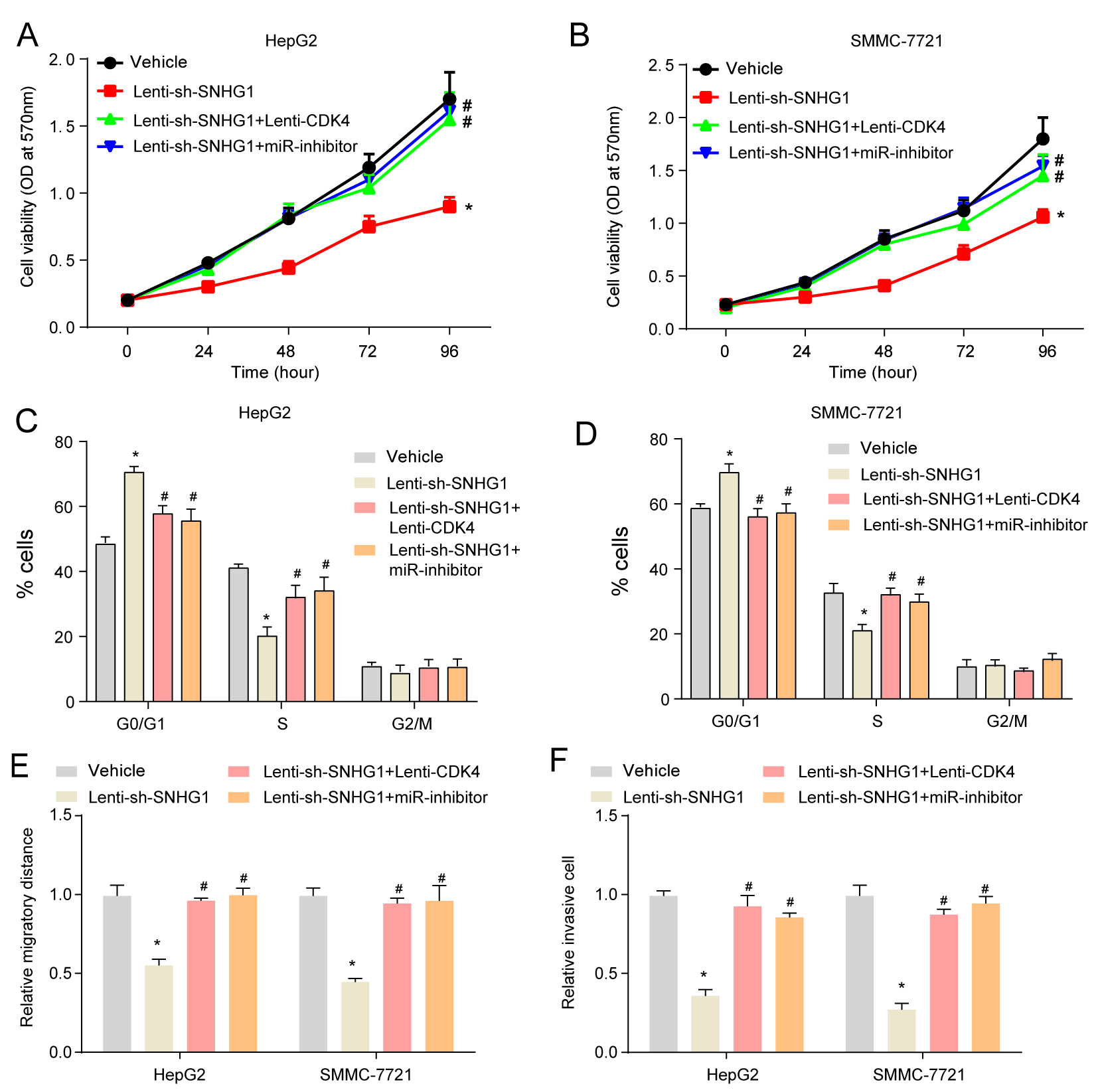

Supplement: Supplementary file 7 — Supplementary Figure S4 [file 41419_2020_3031_MOESM7_ESM.tif]

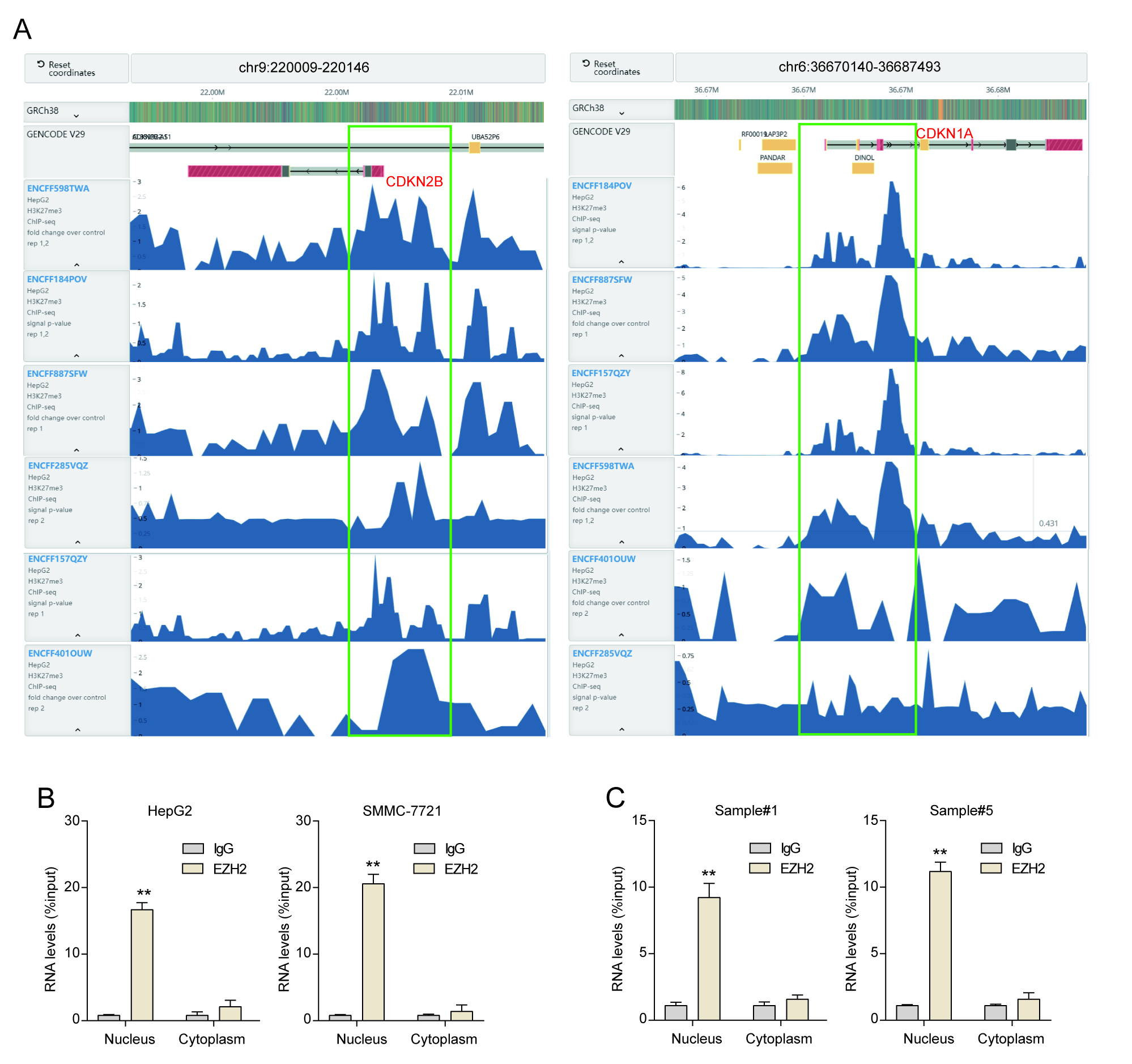

Supplement: Supplementary file 8 — Supplementary Figure S5 [file 41419_2020_3031_MOESM8_ESM.tif]

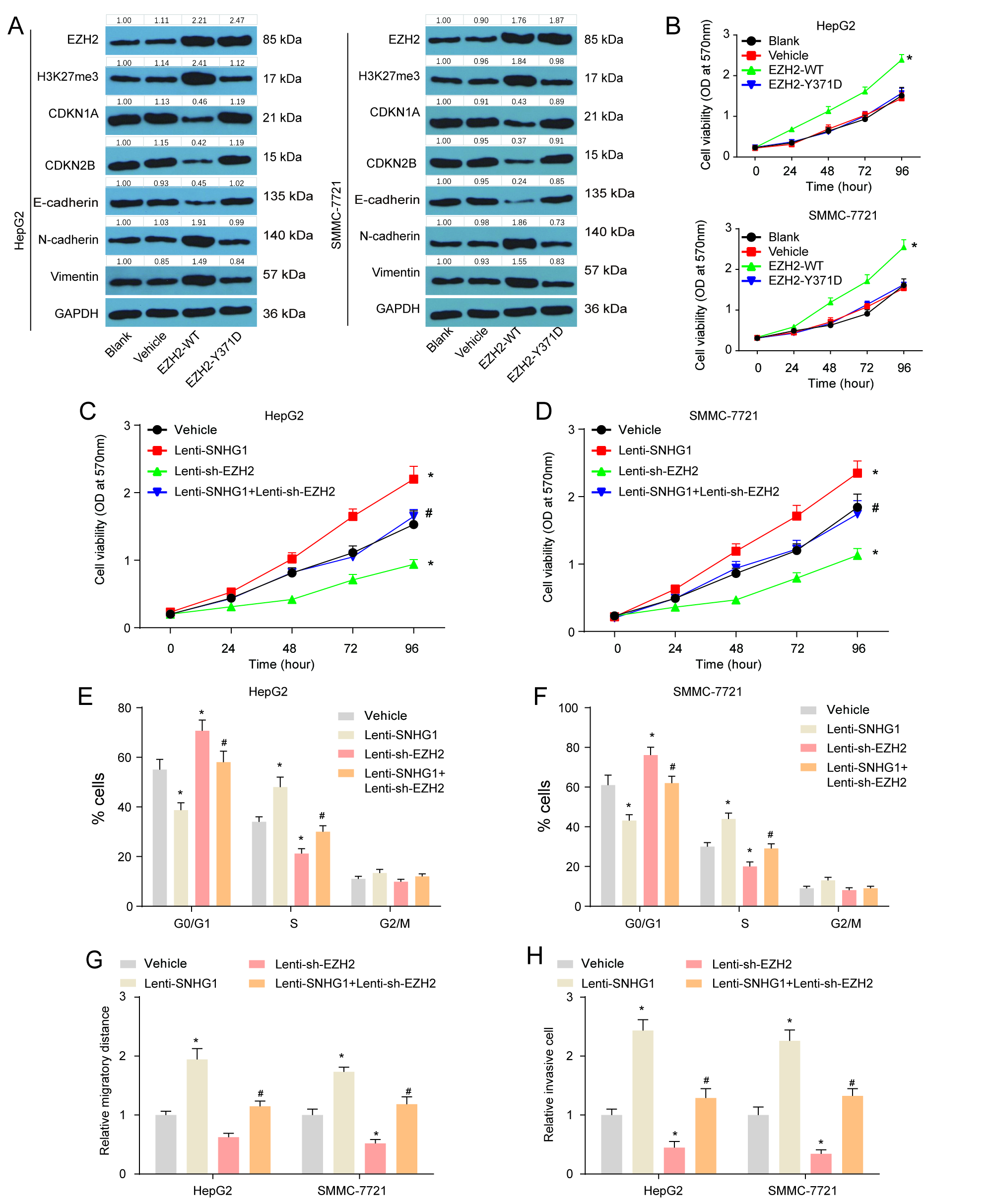

Supplement: Supplementary file 9 — Supplementary Figure S6 [file 41419_2020_3031_MOESM9_ESM.tif]

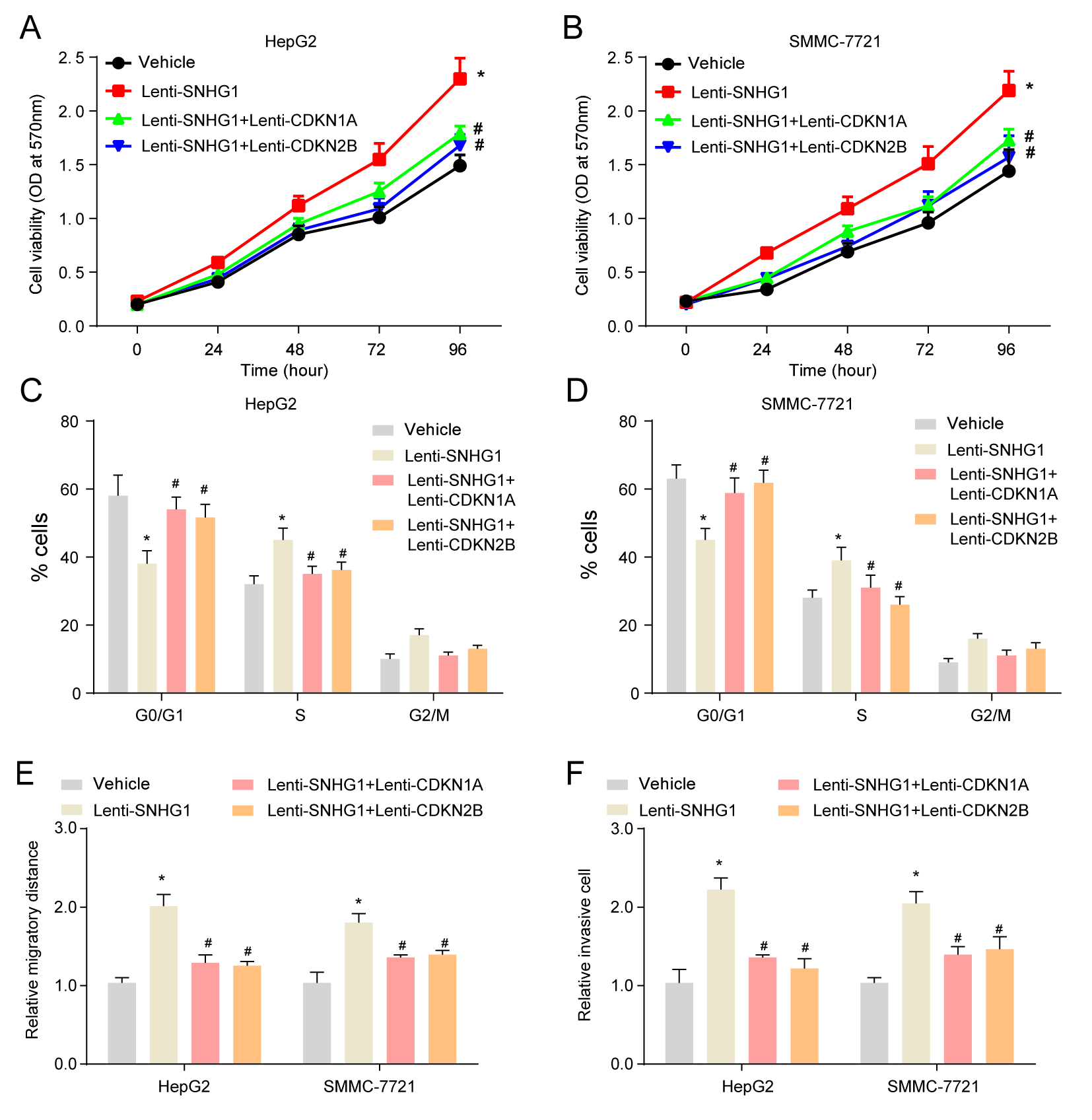

Supplement: Supplementary file 10 — Supplementary Figure S7 [file 41419_2020_3031_MOESM10_ESM.tif]

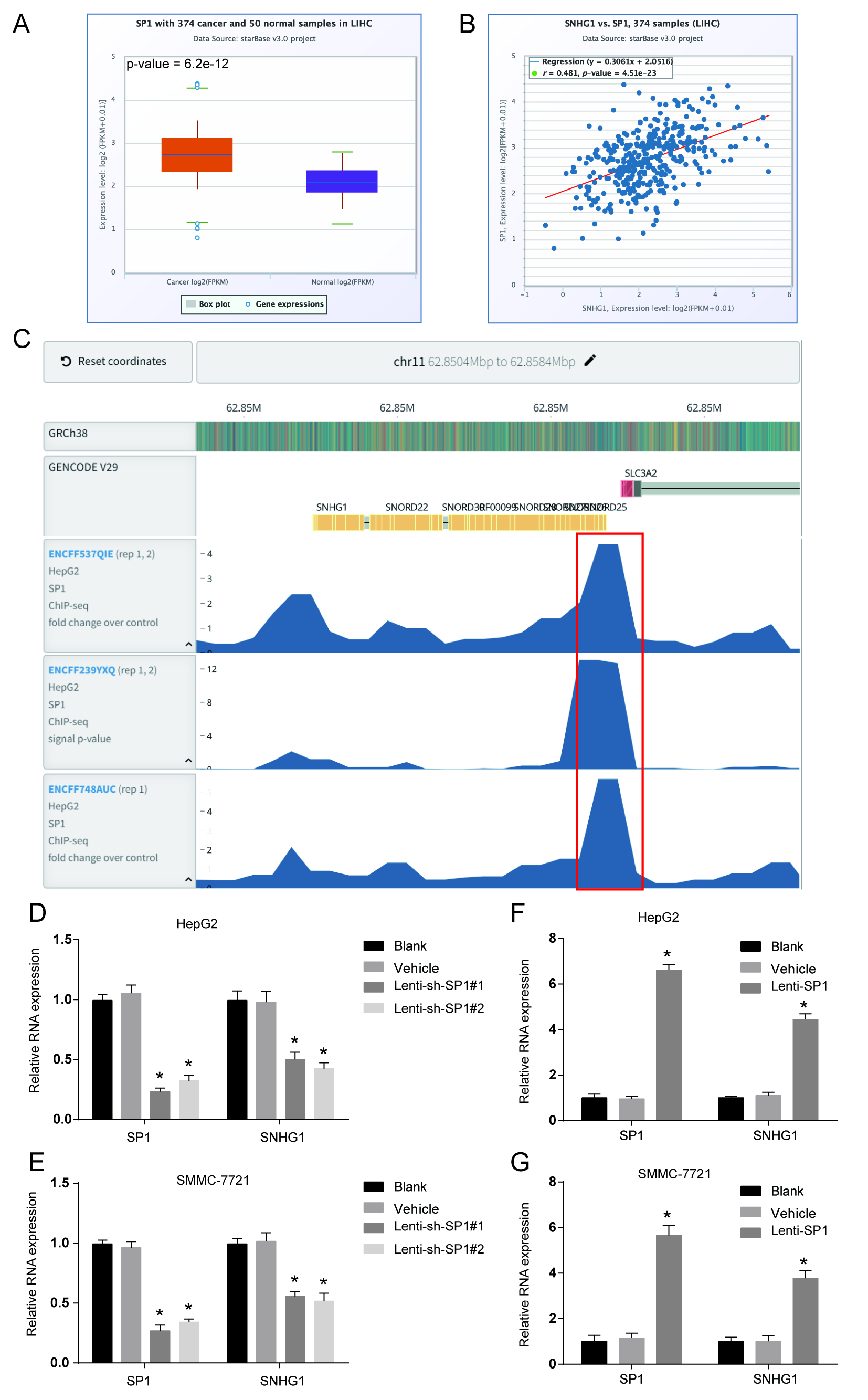

Supplement: Supplementary file 11 — Supplementary Figure S8 [file 41419_2020_3031_MOESM11_ESM.tif]
