# Supplementary material for: Epigenetic silencing of CDKN1A and CDKN2B by SNHG1 promotes the cell cycle, migration and epithelial-mesenchymal transition progression of hepatocellular carcinoma
Source: Cell Death Dis. 2020 Oct 2;11(10):823. doi: 10.1038/s41419-020-03031-6 (PMC7532449; doi:10.1038/s41419-020-03031-6)
Supplement: Supplementary file 12 — Supplementary Materials and Methods [file 41419_2020_3031_MOESM12_ESM.docx]

**Supplementary Materials and methods**

**Microarray analysis**

Data of lncRNAs, mRNAs and miRNAs expressions in normal and HCC tissues were downloaded from GEO (https://www.ncbi.nlm.nih.gov/geo/) (GSE115018 and GSE115016). Gene expression data was processed through the Limma package of the R software. Data normalization in each array was conducted by quantile normalization procedure. LncRNAs, mRNAs and miRNAs were considered to be significantly differential expressed if they reached the standards that *P* value < 0.05 (BH method) and |log_2_FC| > 1.

**Gene set enrichment analysis (GSEA)**

To determine specific pathways of enriched genes, differential expression data was obtained from the microarray analysis and was submitted to GSEA V3.0. The local Human-KEGG database and log2 ratio of class method were utilized in implementation of KEGG enrichment analysis. KEGG pathways with notable enrichment results in HCC in comparison with normal tissues were demonstrated based on normalized enrichment score (NES), gene ratio and *P* value. DOSE, clusterprofiler, ggplot2, ggjoy, GSEABase and annotate packages in R software were exerted to visualize the dysregulated signaling pathways in HCC compared with adjacent normal control.

**Co-expression network analysis**

Aiming to clarify the potential relationship between lncRNAs and mRNAs with different expression levels in HCC, co-expression networks of lncRNAs-mRNAs were established based on Pearson Correlation Coefficient (PCC). The cutoff between lncRNAs and mRNAs was set as 0.7 for PCC, and *p* value less than 0.05 was considered to be meaningful. The paired lncRNAs and mRNAs matching these cutoff conditions were selected and regarded as nodes in the network, and the relationship between these nodes were demonstrated by the PCC value. R package of psych was applied to produce node file and edge file, which were utilized to visualize the co-expression network in Cytoscape (v. 3.6.0).

**Target predictions of lncRNA and mRNA**

MiRNAs targeted by lncRNAs or mRNAs were predicted according to TargetScan database version 7.2 (http://www.targetscan.org/vert_72/) and miRanda (http://www.microrna.org/microrna/home.do). Those miRNAs that co-targeted by the certain lncRNA and mRNA were cross-checked in GSE115016 where the miRNA profiles of the same patients in GSE115018 were available. The final result was displayed by Venny 2.1.0 (http://bioinfogp.cnb.csic.es/tools/venny/).

**Quantitative real time polymerase chain reaction (qRT-PCR)**

RNA extraction from tissues or cells was performed by TRIzol reagent (Invitrogen, Carlsbad, CA, USA) under the instruction of the manufacturer. After that, reverse transfection of total RNA into complementary DNA (cDNA) was conducted by the M-MLV Reverse Transcriptase (Thermo Fisher Scientific, Waltham, MA, USA) in according with the direction of manufacturer. Specifically, for quantification of miRNAs, miRNA was reverse-transcribed using the Mir-XTM miRNA First-Strand Synthesis Kit (TaKaRa, China) following the protocol. ABI 7500 RT-PCR System (Applied Biosystems, CA, USA) with SYBR Premix Ex Taq (Takara, China) was used to perform qRT-PCR based on the guidance of manufacturer. The sequences of primers were demonstrated in Supplementary Table S2. The relative RNA expressions were quantified using the $2^{(-\Delta\Delta C_{t})}$ method. Each sample was independently analyzed in triplicate.

**Western blot**

Total proteins were extracted from cells or tissues, and protein concentration was estimated by a Bradford protein assay kit (Beyotime, Shanghai, China). Approximate 50 μg of protein was separated using 10% sodium dodecyl sulfate polyacrylamide gel electrophoresis (SDS-PAGE) and electro-transferred onto nitrocellulose membranes. 5% fat-free dry milk in tris-buffered saline supplemented 0.1% Tween-20 (TBST) were added to the membranes for 1.5 hours at room temperature (RT) in order to block non-specific antigen. Thereafter, the membranes were incubated with the primary antibodies against CDK4 (1:2000, Abcam, ab137675), CCND1 (1:1000, Abcam, ab134175) CDKN1A (1:2000, Abcam, ab109520), CDKN2B (1:2000, Abcam, ab53034) Rb (1:1000, Abcam, ab17512), p-Rb (1:1000, Abcam, ab47474), E2F1 (1:2000, Abcam, ab112580), E-cadherin (1:1000, Abcam, ab1416), N-cadherin (1:1000, Abcam, ab76057), Vimentin (1:2000, Abcam, ab92547) and GAPDH (1:5000, Abcam, ab9485) at 4°C overnight. After incubation, the membranes were washed by TBST and then incubated with secondary IgG-HRP (1: 5000, Abcam, ab7090/ab205719) for 2 hours at RT. Enhanced chemiluminescence reagent (Pierce, Rockford, IL, USA) was used to visualize the protein bands.

**MTT assay**

Cell Proliferation Reagent Kit I (MTT) (Roche, Basel, Switzerland) was employed to monitor the viability of cells. Cells were plated in 96-well plates and all the process were carried out under the manufacture’s protocol. Cell proliferation was observed and evaluated every 24h. Absorbance was recorded at 570nm.

**Wound healing assay**

Cell migration was determined by wound healing assay. Transfected cells were planted into a 24-well plate and then cultured to 90% confluence. Plastic pipette tip was used to produce a wound in the cell monolayer. Then, cells were washed by PBS. Loose cells were removed and incubated for 36 hours at 37°C. Images were recorded at 0 hours and 48 hours, respectively. Cell migratory distance was determined by Image-Pro Plus software (version 3.0; Media Cybernetics).

**Transwell invasion assay**

To identify cells invasive ability, 1 × 10^5^ cells were seeded in the top chamber of transwell plates (8.0 μm, polycarbonate membrane, Chemicon, Temecula, CA, USA). 2 mg/ml Matrigel was inserted. The lower chamber was added with DMEM supplemented with 10% FBS. Cells in the top-chamber were incubated for 48 hours. Cotton swab was used to remove cells that did not invade through the polycarbonate membrane, while those invaded through the polycarbonate membrane were fixed with 20% methanol. Fixed cells were stained with 0.2% crystal violet. Cells were counted by using a microscope (Olympus, Tokyo, Japan) to determine the cell invasive ability.
